# Identification of urine protein biomarkers with the potential for early detection of lung cancer

**DOI:** 10.1038/srep11805

**Published:** 2015-07-02

**Authors:** Hongjuan Zhang, Jing Cao, Lin Li, Yanbin Liu, Hong Zhao, Nan Li, Bo Li, Aiqun Zhang, Huanwei Huang, She Chen, Mengqiu Dong, Lei Yu, Jian Zhang, Liang Chen

**Affiliations:** 1 School of Life Science, Tsinghua University, Beijing 100084, China; 2 National Institute of Biological Sciences, Beijing 102206, China; 3 Xijing Hospital, Xi’an 510060, China; 4 General Hospital of the People’s Liberation Army, Beijing 100853, China; 5 Beijing Tongren Hospital, Capital Medical University, Beijing 100730, China; 6 National Institute of Biological Sciences, Beijing, Collaborative Innovation Center for Cancer Medicine, Beijing 102206, China

## Abstract

Lung cancer is the leading cause of cancer-related deaths and has an overall 5-year survival rate lower than 15%. Large-scale clinical trials have demonstrated a significant relative reduction in mortality in high-risk individuals with low-dose computed tomography screening. However, biomarkers capable of identifying the most at-risk population and detecting lung cancer before it becomes clinically apparent are urgently needed in the clinic. Here, we report the identification of urine biomarkers capable of detecting lung cancer. Using the well-characterized inducible Kras (G12D) mouse model of lung cancer, we identified alterations in the urine proteome in tumor-bearing mice compared with sibling controls. Marked differences at the proteomic level were also detected between the urine of patients and that of healthy population controls. Importantly, we identified 7 proteins commonly found to be significantly up-regulated in both tumor-bearing mice and patients. In an independent cohort, we showed that 2 of the 7 proteins were up-regulated in urine samples from lung cancer patients but not in those from controls. The kinetics of these proteins correlated with the disease state in the mouse model. These tumor biomarkers could potentially aid in the early detection of lung cancer.

Lung cancer is the leading cause of cancer-related disease worldwide, causing 1.8 million deaths per year worldwide as of 2008^1^,^2^,^3^. Despite advances in multimodal therapies, the prognosis of lung cancer remains disappointingly poor.

Recently, the National Lung Screening Trial (NLST)^4^ demonstrated a significant relative reduction in mortality in high-risk individuals with low-dose spiral computed tomography (LDCT) screening. A similar conclusion was reached by the Early Lung Cancer Action Program (ELCAP)^5^. Unfortunately, in addition to its high cost, LDCT scanning has a high rate of false positives. Moreover, a potential concern about LDCT scanning is that an increased risk of lung cancer is associated with radiation exposure^6^. Thus, effective, alternative methods of large-scale screening are urgently needed in the clinic.

Cancer is a genetic disease that develops through the progressive accumulation of activating alterations to growth-promoting oncogenes and inactivating alterations to tumor-suppressor genes. These changes result in a marked difference in gene and, therefore, protein expression between normal and cancerous cells, some of which can be captured in peripheral body fluids and analyzed to determine the status of a tumor *in vivo*. Studies of other forms of cancer have shown that the use of diagnostic tests based on body fluid protein biomarkers significantly improved patient survival rates. One clinical study of Hepatocellular carcinoma (HCC) showed that a surveillance program consisting of measurements of AFP levels and a liver ultrasound performed every 6 months resulted in a 37% reduction in mortality^7^. Strikingly, monitoring AFP levels alone has been observed to result in a significant increase in 5- and 10-year tumor-free survival^8^. Currently, intensive research is being conducted to identify biomarkers capable of identifying the populations most at risk for a disease and of detecting the disease before it becomes clinically apparent. Unfortunately, no biomarkers are available for clinical use for efficiently screening patients at a high risk of developing lung cancer.

Peripheral biofluids, such as serum, are preferred source of samples for identifying biomarkers for the early detection of tumors^9^. Research has suggested that the early detection of lung cancer could potentially be achieved through the analysis of biomarkers in tissue samples from within the respiratory tract, including sputum^10^, saliva^11^, nasal/bronchial airway epithelial cells^12^ and exhaled breath condensate^13^. Recently, urine has attracted increased attention as a biospecimen that may be useful for the identification of cancer biomarkers. Urine is protein-rich and contains approximately 3,000 detectable protein species. Moreover, urine proteins are stable because the secreted proteins are mature, and urine is easy to collect in large volumes in a noninvasive manner (reviewed in^14^).

Despite advances in technology and intensive research efforts, no molecular biomarker capable of identifying lung cancer in the early stages has been found that is suitable for clinical use. The lack of a validated biomarker for the early detection of lung cancer, in spite of intensive research, is partly due to the drawbacks of the methods typically used in the search for biomarkers. A high level of noise in proteomic assays results from differences in genetic backgrounds, environments and lifestyles between patients and matched controls, and this noise interferes with the successful identification of disease-associated biomarkers^15^. Genetically engineered mouse models (GEMMs) harboring the genetic aberrations of human tumors phenocopy human malignancies and recapitulate the clinical course of tumor development in patients. Therefore, combining the advantages of GEMMs with those of human samples should increase the chances for the successful identification of biomarkers for the early detection of lung cancer^16^,^17^.

Here, we report the detection of proteomic changes in urine samples from tumor-bearing genetically engineered mice (GEMs) compared with matched controls. Similarly, lung cancer patients showed marked differences in the urine proteome compared with matched controls. By comparing these proteomic data, we identified potential biomarkers that are common to both tumor-bearing mice and lung cancer patients. Moreover, we validated several of these biomarkers in an independent cohort of lung cancer patients. Using the doxycycline-inducible mutant Kras mouse model of lung cancer, we showed that urine ceruloplasmin (CP) levels increased as tumors progressed and decreased as tumors regressed.

## Materials And Methods

### Mouse work

All mice were housed in a pathogen-free environment at the National Institute of Biological Sciences, Beijing (NIBS). All experimental protocols were approved by the Institutional Committee for Animal Care and Use at NIBS. All animal work was performed in accordance with the approved protocol.

### Ethics statement

The protocol for human research was approved by Xijing Hospital at the Fourth Military Medical University in Xi’an, China, by the Fuzhou Pulmonary Hospital in Fujian, and by the Institutional Committee at the NIBS. Written informed consent was obtained from every patient who donated tissue. All work was performed in accordance with the approved protocol.

### Patients and control subjects

Lung cancer patients enrolled in this study were from Xijing Hospital of the Fourth Military Medical University in Xi’an, China and from the Fuzhou Pulmonary Hospital of Fujian. Written informed consent was obtained from all patients who donated urine and serum samples. Controls were age- and gender-matched healthy subjects.

### Urine collection

Mouse urine was collected using metabolic cages (TECNIPLAST, 3700M022, Italy), and centrifuged at 10,000 rpm for 5 min at 4 °C to remove fecal debris. Protease inhibitors (Roche, 4693132001, Switzerland) were immediately added to the urine samples, which were then aliquoted into sterile centrifuge tubes and stored at −80 °C for future analysis. A total of 50 ml of human morning midstream urine was collected and filtered through 0.45-μm filters. A protease inhibitor was applied immediately after filtration, and the human urine samples were stored at −80 °C until use.

### Antibody production

We used the DNASTAR program to select immunogenic peptide antigens for the production of rabbit polyclonal antibodies against human and mouse CP. Sera from rabbits immunized with the following four peptides were used: (ECNKSSSKDNIRGKHVRC, human; EPEKVDKDNEDFQESNRC, NIKTYSEHPEKVNKDNC, and QVDKEDEDFQESNKC; mouse). The sera were validated by western blotting at 1:1,000 dilutions using 293T cells expressing human and mouse CP and control 293T cells. These sera showed high affinity and specificity by western blotting.

### Data analysis

Comparisons between the two groups were made in Microsoft Excel (Microsoft, Redmond, WA, USA) using Student’s t-test. A value of p < 0.05 (*) was considered statistically significant, and value of p < 0.01 (**) was considered highly statistically significant.

## Results

### Combining a genetically engineered mouse model with clinical samples for the successful identification of lung cancer biomarkers

**Figure 1:**
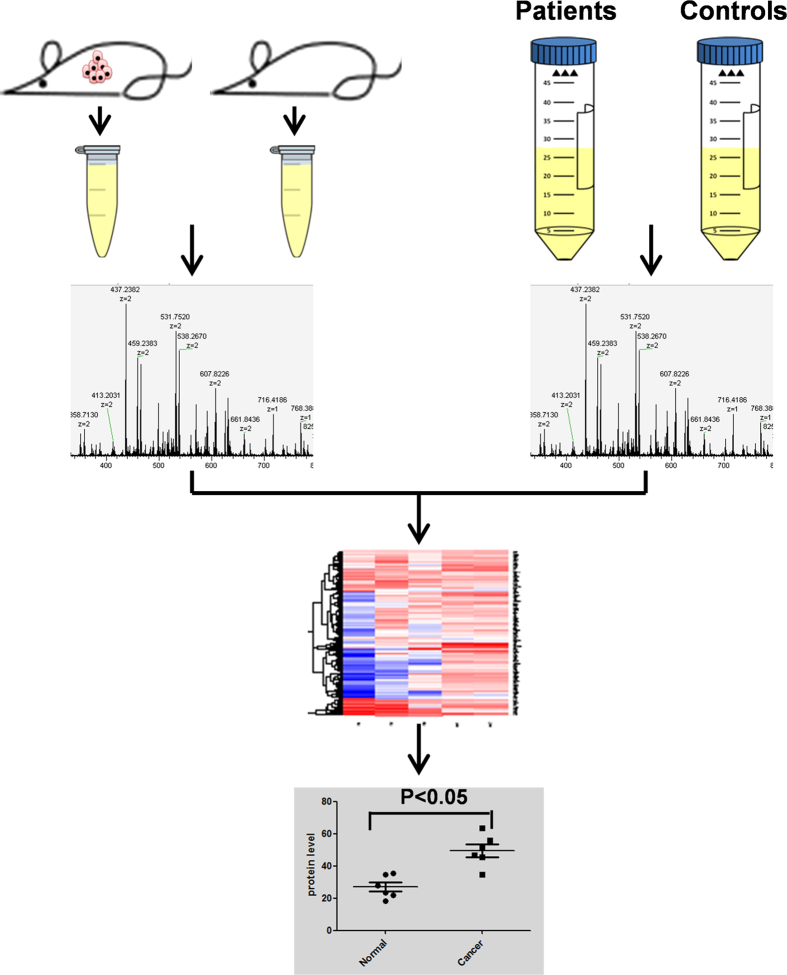
Schematic diagram of the experimental design. Urine samples from paired control and tumor-bearing mice and paired lung cancer patients/healthy controls were subjected to LC-MS analysis. Up-regulated protein candidates common to patients and tumor-bearing mice were identified. Western blotting and ELISA were used to validate the candidate proteins in an independent cohort of clinical samples.

Drastic changes in expression and functioning in oncogenes and tumor suppressor gene are prominent features of tumor^18^,^19^,^20^. A marked alteration in both gene and protein expression between normal and cancerous cells is reflected in alterations of the proteomic profiles of the body fluids of cancer patients^21^,^22^,^23^. Currently, biomarker identification in peripheral body fluids is achieved by comparing proteomic patterns between patients and healthy controls. Unfortunately, differences in genetic backgrounds, environments and lifestyles between healthy individuals and patients result in a high level of noise in proteomic signals, and because of this high noise level, it is difficult to identify protein markers of *in vivo* tumor burden. We hypothesize that only a few up-regulated urine proteins are capable of identifying lung cancer tumor burdens; we termed this group of proteins the urine “signature” of lung tumor burdens. However, compared with the urine of healthy controls, patients’ urine contains not only this signature of up-regulated proteins but also many other proteins that reflect differences in genetic backgrounds, environments and lifestyles. By contrast, GEMMs feature pure genetic backgrounds and strictly controlled environments that minimize noise that obscures the identification of urine biomarkers for lung cancer. By identifying up-regulated proteins that are common to both tumor-bearing mice and patients, we maximize our chance of identifying actual tumor signature proteins in urine. Thus, we propose a procedure for efficient identification of urine biomarkers, shown in the schematic in Fig. 1.

Two controllable mouse model LSL-KRAS^G12D^ mice and TetO- KRAS^G12D^ mice were applied for urine protein profiling. We collected urine, labeled urine proteins with isobaric tags, and quantitatively compared the levels of the proteins by LC-MS. Urine samples from lung cancer patients positive for the *Kras* mutation and matched healthy controls were likewise compared quantitatively. Using this method, up-regulated urine proteins common to both the tumor-bearing mice and lung cancer patients were identified. The identified protein biomarkers were then verified in urine samples from an independent cohort of human subjects.

### Proteomic profiling of LSL-KRAS^G12D^ and TetO- KRAS^G12D^ mice urine

**Figure 2:**
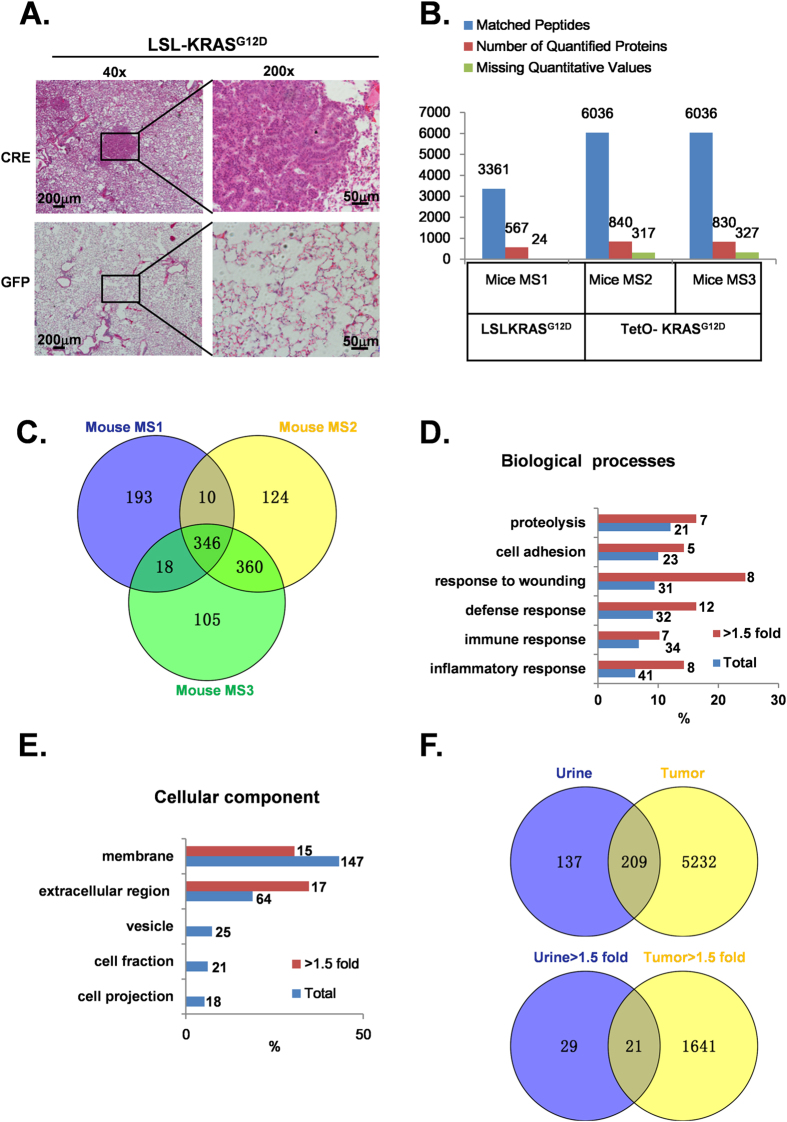
Proteomic comparison of urine samples derived from LSL-KRAS^G12D^ and TetO- KRAS^G12D^ mice. **A**. H&E staining was performed on Ad-CRE (upper panel) and Ad-GFP (lower panel) virus-treated LSL-KRAS^G12D^ mouse lung tissues 3.5 months post-induction (scale bars represent 200 μm and 50 μm in X40 and X200 magnification, respectively). **B**. Overview of the proteins identified and quantified on two mouse models. The numbers of matched peptides, quantified proteins and proteins missed quantification value are shown above the bar graph. **C**. Comparison of 3 mouse proteomic data reveals 346 overlapped proteins. **D**,**E**. Classification of the common proteins and up-regulated (>1.5-fold change) proteins in mice urine by biological process and sub-cellular distribution. The X-axis indicates the percentage of proteins associated with each term; the numbers of proteins associated with each term are presented on the right. **F**. 209 protein was found to be common both in lung tumor tissue and mice urine. Of the 209 common proteins, 21 proteins were up-regulated.

*Kras* mutations, especially the G12 mutation, are found in a significant portion of lung cancer patients and are biomarkers for poor prognoses^24^. Therefore, it is of great importance to identify an early diagnostic marker for this type of lung cancer. In our study, two well-characterized controllable mouse models LSL-KRAS^G12D^ and TetO- KRAS^G12D^ were used for urine protein profiling. Tumor burden was induced by nasal inhalation of a single dose (10^6^ PFU) of Ad-CRE in LSL-KRAS^G12D^ mice. Consistent with earlier reports, we found that tumor nodules were macroscopically visible in all mice 3.5 months post Ad-CRE virus infection^25^,^26^. Histological examination showed a solid architecture and moderate, atypical nuclei in these tumors. Atypical adenomatous hyperplasia, papillary adenoma, and adenocarcinoma were evident (Fig. 2A, upper panel). The tumors were small in size (<0.5 cm) and had not metastasized to lymph nodes and distal organs, both of which findings were indicative of cancer at an early stage. By contrast, Ad-GFP-treated mice showed no phenotypic changes (Fig. 2A, lower panel). Based on these results, we collected urine for proteomic profiling 3.5 months after Ad-CRE or Ad-GFP treatment. To avoid possible limitation associated with LSL-KRAS^G12D^, we also used another mouse model TetO- KRAS^G12D^ for urine protein profiling. Transgenic TetO- KRAS^G12D^ mice were crossed with CC10rtTA mice to generate bi-transgenic CC10rtta; TetO- KRAS^G12D^ mice. These mice were fed a doxycycline diet beginning at 2 months of age. Consistent with earlier reports, after 20 days of doxycycline diet administration we observed mice with panting and hunched postures. These mice were sacrificed and confirmed to present with poorly differentiated lung adenocarcinoma^27^ (Supplementary Figure 1A, 1B). Urine before and 20 days after doxycycline feeding was collected. We then applied iTRAQ (Isobaric tag for relative and absolute quantitation)/LC-MS/MS to analyze the dis-regulated proteins. Successful protein identification and quantification were defined as matching at least one unique peptide to each protein with 95% confidence and p < 0.05. A total of 3361 and 6036 peptides matched to 591 and 1157 proteins were identified in two mouse models. On average, 5 peptides were utilized for the identification of each protein. Among these identified proteins, 569, 840 and 830 proteins were successfully quantified in three independent biological replicates (Fig. 2B). Interestingly, we found 346 proteins in common among the three cohorts, with 50 of them up-regulated (>1.5-fold change) in tumor-bearing mouse urine (Fig. 2C). Proteolysis, cell adhesion, response to wounding, defense response, immune response, inflammatory response (p < 0.05) were the six biological processes that were most significantly enriched both in terms of the total number of proteins and the up-regulated proteins (Fig. 2D, Supporting Information Tables 1, 2). This result is consistent with the finding that the proteome of lung cancer cells is altered to enable the cancerous cell to exhibit a pro-growth effect (the enriched pathways are detailed in Supporting Information Tables 3, 4). Next, we classified the sub-cellular localization of the common proteins and the up-regulated proteins in both of the cohorts. As expected, the largest proportion of the urine proteins were secretary proteins; 43% of the proteins were found in cell membranes (p < 0.05), and another 18% were found in extracellular regions (p < 0.05) (Fig. 2E, Supporting Information Tables 5, 6).

The above results suggest that the urine proteins are most likely derived from cancer cells. To confirm our hypothesis, we harvested the lung cancer nodules and normal lung tissue from the mice and performed proteomic analyses for correlation analyses. Interestingly, we found that among the 5441 proteins detected in the lung cancer modules, 209 were also present in urine and accounted for 66% of the total urine proteins (Fig. 2F). Moreover, the proteins up-regulated in the lung cancer nodules (compared to peritumoral tissues) included 21 protein species that were also found to be up-regulated in the urine of tumor-bearing mice (compared with their non-tumor-bearing sibling controls; Fig. 2F). Among these 21 proteins, most were secreted proteins.

Therefore, our results demonstrated that the inducible mouse lung cancer tumor model could facilitate the identification of potential urine biomarkers for the early diagnosis of lung cancer.

### Proteomic profiling of urine from human patients with *Kras* mutation-positive lung cancer

**Figure 3:**
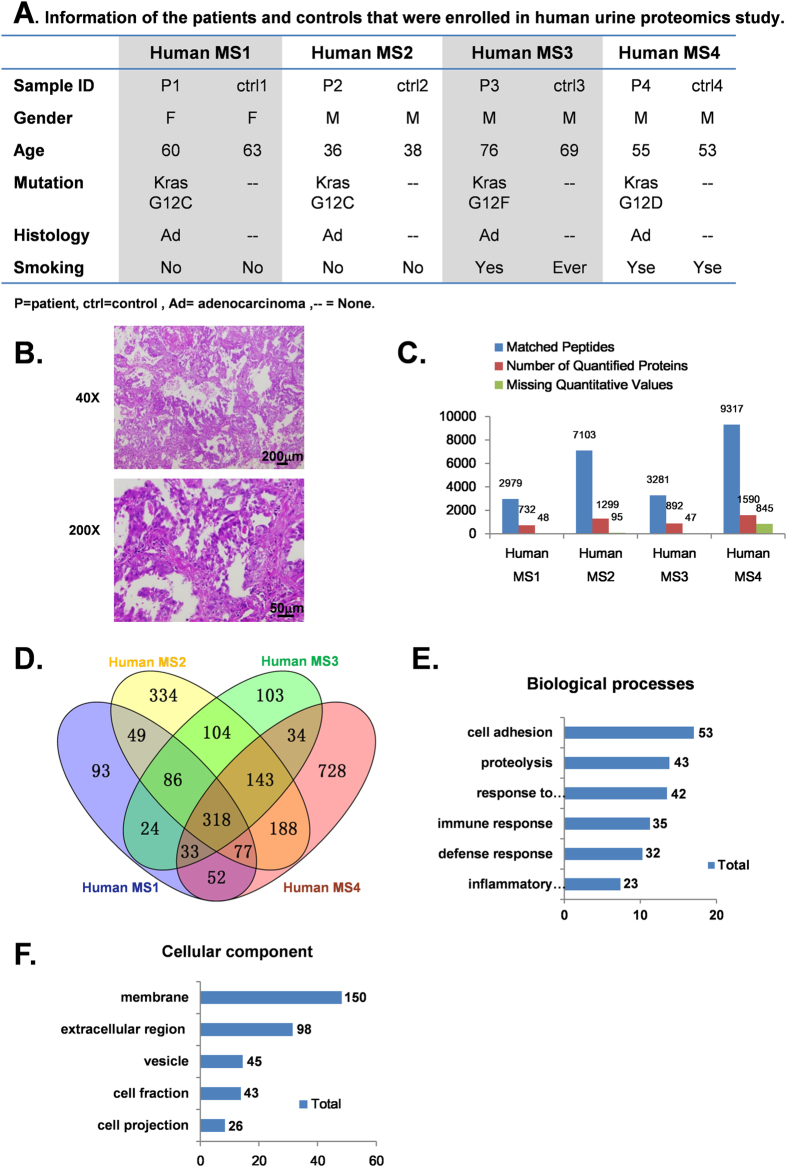
Proteomic alterations in urine samples of lung cancer patients compared with age- and gender-matched healthy controls. **A**. Demographic information for the patients and healthy controls that were enrolled in our human urine proteomics study. **B**. Overview of iTRAQ LC/MS identification and quantification efficiency of the clinical urine samples. **C**. Histology shows lung adenocarcinoma from 1 patient. Scale bars represent 200 μm and 50 μm at X40 and X200 magnification, respectively. **D**. A total of 732, 1299, 845, and 1590 proteins were successfully quantified in 4 independent proteomic analyses. The intersection shows 318 proteins that were identified in four groups simultaneously. Of the 318 proteins, 24 were up-regulated (>1.5-fold change). **E**,**F**. Classification of the common proteins in human urine by biological processes and sub-cellular distribution (p < 0.05). The X-axis indicates the percentage of protein associated with term; the numbers of proteins included in each group are presented on the right.

Although the genetically engineered KRAS^G12D^ mouse model represents a useful platform for identifying biomarkers for the early detection of lung cancer, inter-species differences necessitate validating urine proteins whose levels are altered in these mice in human lung cancer patients. Thus, we collected morning midstream urine from 4 lung cancer patients positive for the *Kras* mutation and from 4 age- and gender-matched healthy controls (detailed information on the patients and controls is provided in Fig. 3A). Pathological analyses revealed poorly differentiated lung adenocarcinomas at various stages (a typical H&E staining result shown in Fig. 3B). We identified 2979, 7103, 3281, and 9317 peptides matching 780, 1394, 939, and 2435 protein species, respectively, for the 4 pairs of patient/control urine samples (Fig. 2C). A total of 318 proteins overlapped in the 4 groups (Fig. 3D), with 24 of them up-regulated (>1.5-fold change). GO analysis based on biological processes and cellular component revealed patterns similar to those observed in the mouse urine (Fig. 3E, Supporting Information Tables 7), (Fig. 3F, Supporting Information Tables 8).

### Up-regulated urine proteins common to both mice and humans

**Figure 4:**
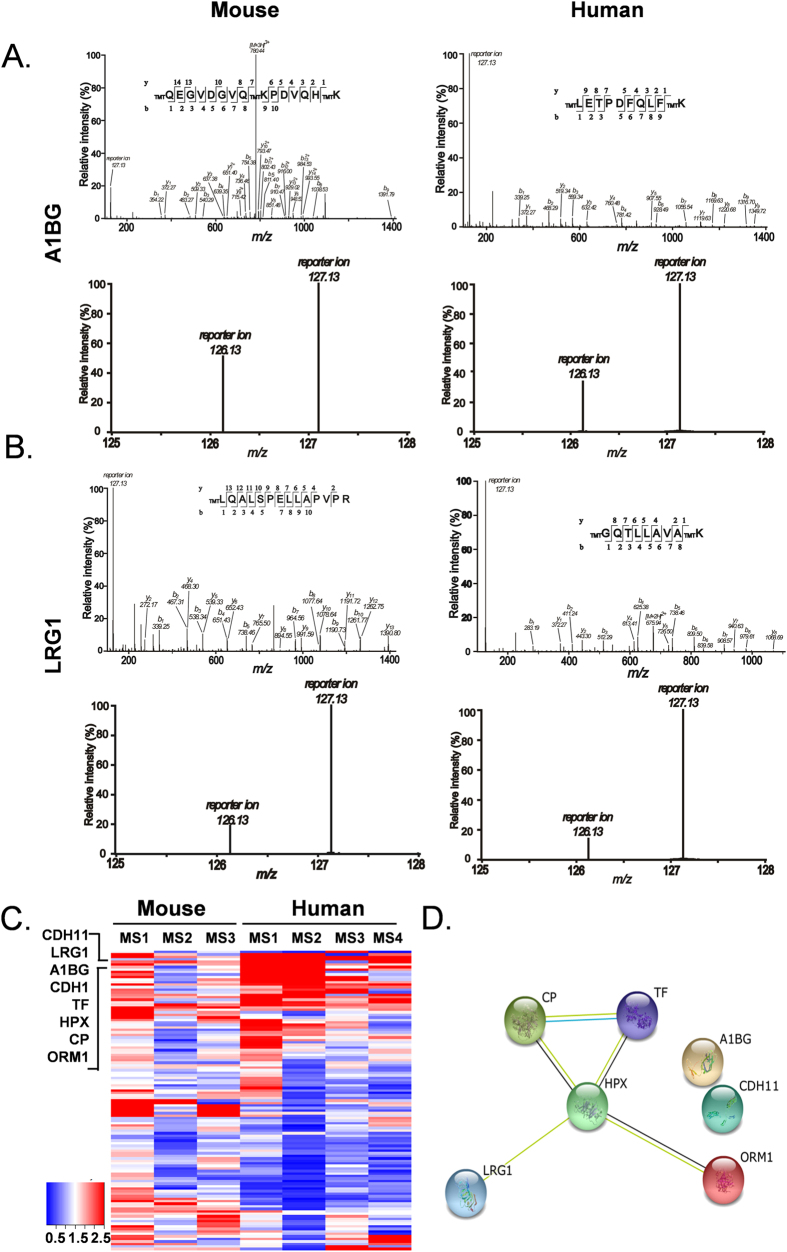
Bioinformatics analysis of the 7 commonly up-regulated proteins. **A**,**B**. MS/MS spectra representing A1BG and LRG1 identified in mouse (left panel) and human (right panel) urine. **C**. Heatmap of 119 common proteins between 3 groups of mice and 4 groups of human urine proteomic analysis. **D**. STRING analysis shows the interaction network of the 7 up-regulated proteins.

Based on our mass spectrometry data, A1BG and LRG1 were among the most markedly up-regulated proteins in the urine samples from tumor-bearing mice. Both proteins were likewise up-regulated in the urine of lung cancer patients (Fig. 4A,B). Interestingly, these two proteins have previously been reported to be potential biomarkers of lung cancer^28^. This finding suggested that the up-regulated urine proteins common to both the tumor-bearing mice and the lung cancer patients were potential biomarkers.

In our set of samples used to identify proteins commonly up-regulated in the urine samples of patients (4 pairs of patients/healthy controls) and tumor-bearing mice (6 pairs of tumor-bearing/control mice), a total of 119 proteins appeared in both human and mouse urine. The heatmap of these 119 overlapping proteins, based on 127/126 ratios in seven iTRAQ mass spectrum analyses, revealed a set of up-regulated proteins common to both patients and tumor-bearing mice: TF(serotransferrin), A1BG(α-1B-glycoprotein), HPX(Hemopexin), CP(ceruloplasmin), LRG1(Leucine-rich-α-2-glycoprotein), CDH11(Isoform 2 of Cadherin-11), and Orm1(ORM1 α-1-acid glycoprotein 1) (Fig. 4C). STRING analysis of these 7 potential candidates demonstrated interactions between Cp, Hpx, Trf, Orm1, and Lrg1 (Fig. 4D). Table 1 provides detailed information about these proteins, including the numbers of confidently matched peptides used for identification and quantification, the cancer/control ratio for each, and their biological functions.

### Validation of candidate biomarkers in the urine of lung cancer patients

Having determined the set of potential protein biomarkers, we validated them in another cohort of patients and population controls.

CP has previously been reported to be increased in the serum of lung cancer patients^29^. Moreover, higher urine levels of this protein were observed in bladder cancer patients compared with patients with hernias and urinary tract infections/hematuria^30^. However, the detection of CP in the urine of lung cancer patients has not been reported. Thus, we tested whether CP was up-regulated in the urine of lung cancer patients using rabbit anti-mouse and anti-human antibodies generated against CP (Supplementary Figure 1C, 1D).

**Figure 5:**
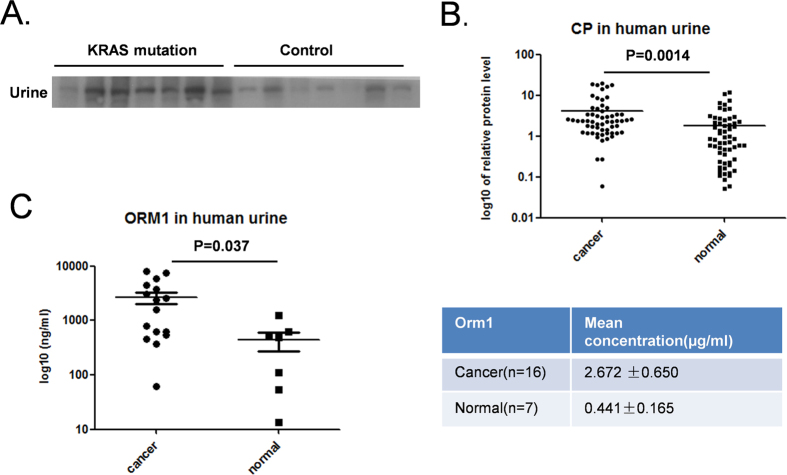
Validation of candidate proteins. **A**. Western blotting analysis of CP in the urine of 7 KRAS mutation lung cancer patients and 7 healthy controls. **B**. Quantification of CP protein levels in urine from 60 cancer patients and 60 healthy controls by western blotting. Band intensities were quantified by ImageJ, p = 0.0014. **C**. Orm1 protein levels were tested in 16 cancer samples and 7 normal controls using a commercial ELISA kit (p = 0.037). The left panel shows the ELISA results of individual samples; the right panel shows the mean concentration ± SD of the two cohorts.

CP levels were measured in sera from a cohort of lung cancer patients harboring the *Kras* mutation and healthy controls. Western blotting revealed a slight increase in serum CP levels in the lung cancer patients (Supplementary Figure 1E), although this difference was not statistically significant. We also collected urine samples from lung cancer patients positive for the *Kras* mutation and from population controls. Consistent with our mass spectrometry data, CP was significantly up-regulated in the cancer patients (Fig. 5A). Because the up-regulation of CP was identified in the urine of Kras mutation-positive lung cancer patients and in the mutant *Kras*-driven mouse model, we investigated whether CP could serve as a urine biomarker for lung cancer patients in general. Urine samples from a total of 60 patients and 60 controls were analyzed, and a significant up-regulation of CP was found in the cancer patients (p = 0.0014; Fig. 5B). By contrast, a total of 36 cancer patients and 18 controls showed a slight increase in CP in the serum (p = 0.063; Supplementary Figure 1F).

From our “signature” set of proteins, we analyzed ORM1 protein levels in a cohort of patients and population controls using a commercially available ELISA kit. Strikingly, we found that the mean urine ORM1 concentration was 2.672 ± 0.65 μg/ml in patients and 0.441 ± 0.165 μg/ml in healthy control subjects (p = 0.037; Fig. 5C).

Demographic information for the patients and control subjects studied for whom urine CP and Orm1 levels were analyzed are provided in Supporting Information Tables 9, 10.

### Ceruloplasmin is a urine marker of lung tumor burden

**Figure 6:**
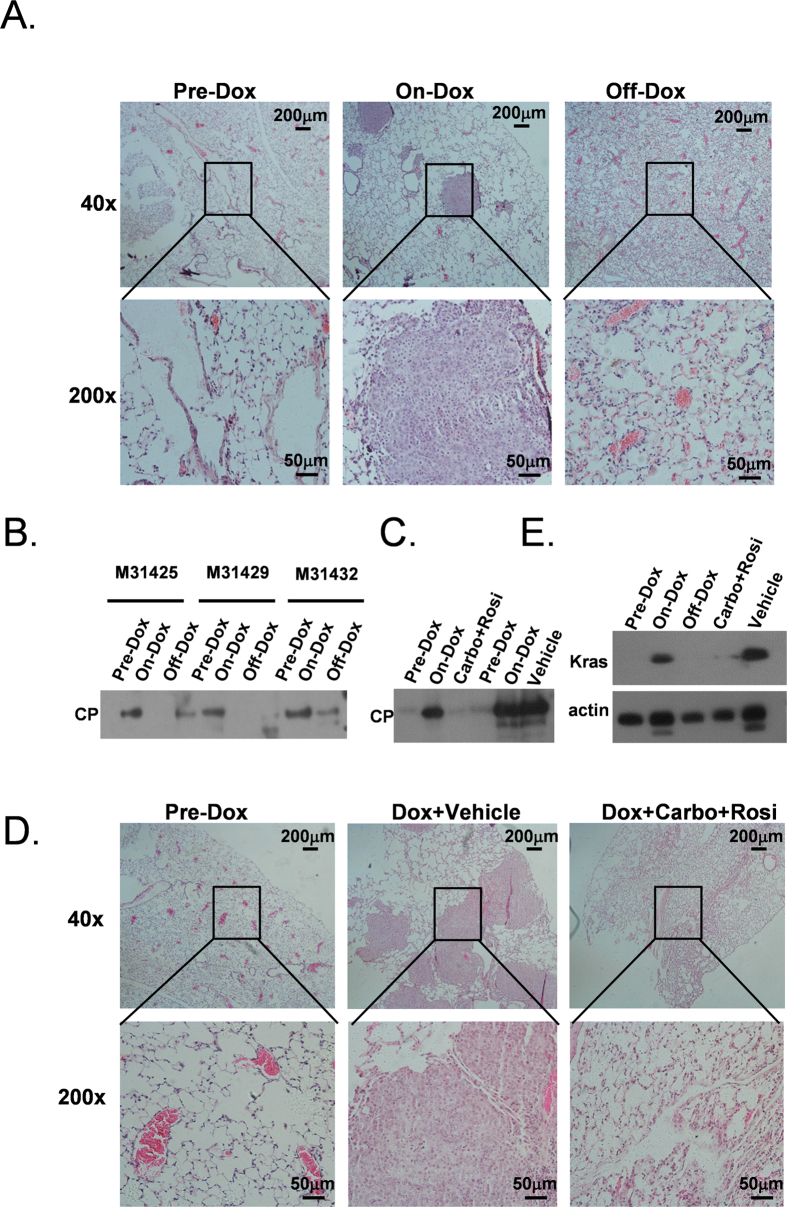
CP levels reflected the *in vivo* tumor burden. **A**. H&E staining of mouse TetO- KRAS^G12D^ lung tissue at three distinct time point. Before doxycycline diet administration (Pre-dox), 20 days after doxycycline diet (On-dox) and 10 days after doxycycline withdrawal (Off-dox). Lung tumors were efficiently induced by the doxycycline diet treatment and regressed in response to doxycycline withdrawal. Scale bars represent 200 μm and 50 μm at X40 (upper panel) and X200 (lower panel) magnification. **B**. Western blotting analysis of CP protein level on bitransgenic mice (numbers M31245, M31429 and M31432) at the three time points. A total of 20 μg protein was loaded. **C**. Western blotting analysis of CP protein levels before and after tumor induction and 20 days after carboplatin and rosigltizone (Carbo + Rosi) or vehicle treatment. **D**. Histology of CC10rtTA;TetO- KRAS^G12D^ mice before and after combination therapy (Dox + Carbo + Rosi) or vehicle (Dox + Vehicle) treatment. **E**. Kras protein level in the condition of doxycycline induction and combination therapy or vehicle treatment.

Tumor biomarkers are expected to be able to indicate the status of a tumor, and therefore fluctuations in concentrations should reflect the progression or regression of a tumor. To validate whether this is true of the protein markers identified in this study, we examined the expression of CP in the TetO- KRAS^G12D^ mouse model. We first carefully monitored the time point of tumor regression on this mouse model. Interestingly, after 10 days of doxycycline withdrawal, the tumors were largely gone (Fig. 6A). Then, we validated whether the CP level corresponded to the tumor burdens in these mice. Urine was collected at three distinct time points: before doxycycline treatment (Pre-Dox), 20 days after doxycycline treatment (On-Dox) and 10 days after doxycycline withdrawal (Off-Dox). Strikingly, fluctuations in CP protein levels in the urine correlated with the tumor burdens in the mice. The CP level increased drastically 20 days after the doxycycline diet treatment. Importantly, the protein level decreased drastically when the doxycycline diet was withdrawn (Fig. 6B). This result demonstrated that the CP levels reflected the tumor burden in mice.

To mimic the clinical setting, we tested whether CP levels changed in response to treatment. Previously, we reported that combination treatment with carboplatin and rosigltizone led to a dramatic regression of Kras-driven tumors^31^. Therefore, we monitored the urine CP level in a cohort of mice prior to doxycycline treatment, after doxycycline treatment and after combination treatment. As expected, we found that the urine CP level increased from barely detectable to a high level in response to doxycycline treatment. Strikingly, we found that combination treatment resulted in almost undetectable level of CP in the urine in these mice. In comparison, the CP levels in the urine of the mock-treated mice remained high (Fig. 6C). Correspondingly, histological analysis found a drastic tumor regression in the combination treatment group (Fig. 6D). Kras protein level in the condition of doxycycline induction and combination treatment was show in Fig. 6E.

## Discussion

Because lung cancer is a disease with a high mortality rate, an efficient method of early diagnosis is urgently needed in the clinic. In this study, using a new strategy, we identified a panel of urine biomarkers of lung cancer. We also validated a portion of these biomarkers in lung cancer patients.

Researchers have attempted to identify urine biomarkers by analyzing human samples. Although biomarkers identified using this strategy would be applicable in clinical settings, several limitations contribute to high noise levels in such proteomic analyses that hinder the successful identification of biomarkers capable of indicating tumor burdens. Human subjects are known to vary greatly in their genetic backgrounds, lifestyles, and environments. By contrast, GEMMs feature pure genetic backgrounds and highly controlled environments. Thus, the noise levels in proteomic analyses of GEM samples are expected to be much lower than those in analyses of human samples. Thus, by combining the advantages of both systems, we were able to identify biomarkers of lung cancer.

In lung cancer research, in contrast with research on bladder cancer or renal cell carcinoma, urine is seldom examined in the search for biomarkers. However, recent work has shown that urine may be of use in the search for biomarkers of lung cancer^32^. In this study, we have shown that some protein species up-regulated in lung cancer nodules are also present at higher concentrations in the urine of lung cancer patients.

Interestingly, A1BG and LRG1 were reported to be secreted by the A549 cell line and were found to be elevated in the serum of lung cancer patients^28^. The relative concentration of genetic variants of Orm1 were increased in lung cancer patients as well^33^,^34^. Moreover, the incidence of cancer was reported to be positively associated with serum ceruloplasmin (CP) levels; the association was found to be strongest for lung cancer and other cancers related to smoking^29^. The same study also suggested that high serum CP levels in lung cancer may mainly be due to occult cancer. We confirmed that CP levels were elevated in the serum of lung cancer patients; however, in our study, this difference did not reach statistical significance, most likely because of the limited sample size. By contrast, we found that CP levels were markedly elevated in the urine of lung cancer patients from the same cohort of patients and healthy controls. This finding showed that our strategy constituted a powerful method for identifying true biomarkers of disease.

Notably, a significant proportion of the up-regulated urine proteins in lung cancer patients are involved in inflammation, which potentially contributes to the development of lung cancer. Indeed, mutant *Kras*-driven lung cancer features extensive inflammation^35^, and the mutant KRAS in lung cancer activates the NF-κB signaling pathway^36^. That several proteins that we found to be up-regulated are involved in inflammation thus suggested that our urine “signature” may represent true urine biomarkers of mutant Kras-positive lung cancers.

In our study, we concentrated on proteins that were commonly up-regulated in the urine samples of cancer patients and tumor-bearing mice. However, down-regulated proteins may also be suitable biomarkers that could be used in the early diagnosis of lung cancer. We suggest that some markedly down-regulated proteins merit further study.

In conclusion, we propose that combining the use of patient samples with the use of a mouse model represents a highly efficient method of identifying potential biomarkers. Further studies are needed to validate the clinical performance of the urine protein biomarkers reported here.

## Additional Information

**How to cite this article**: Zhang, H. *et al.* Identification of urine protein biomarkers with the potential for early detection of lung cancer. *Sci. Rep.* **5**, 11805; doi: 10.1038/srep11805 (2015).

## Supplementary Material

Supplementary Information

## Figures and Tables

**Table 1:** Detailed information on the 7 up-regulated proteins identified by iTRAQ analysis.

Mouse IPI Accession	Protein description	Mice MS1		Mice MS2		Mice MS3		Human MS1		Human MS2		Human MS3		Human MS4		SD- geo	Function
		No. of pep	CRE/GFP	No. of pep	Pre/On	No. of pep	Pre/On	No. of pep	C 1/N l	No. of pep	C 2/N 2	No. of pep	C 3/N 3	No. of pep	C 4/N 4		
IPI00139788	TF Serotransferrin	32	3.34	88	1.99	88	2	32	2.873	39	4.325	21	2.625	57	6.572	1.54	iron transport
IPI00128484	HPX Hemopexin	13	3.99	33	2.08	33	2.1	13	2.163	15	2.481	9	1.599	23	2.327	1.32	heme transport
IPI00117831	CP Ceruloplasmin	8	3.24	45	2.61	45	2.6	16	2.048	15	1.97	10	2.078	36	2.217	1.20	copper binding, ferroxidase
IPI00138190	CDH11 Cadherin-11	6	1.78	9	1.78	9	1.8	3	1.639	15	1.567	3	1.501	17	1.499	1.08	cell adhesion
IPI00129965	A1BG α-1B-glycoprotein	2	2.13	20	1.9	20	1.9	10	3.011	13	5.392	6	2.317	13	2.405	1.44	plasma glycoprotein
IPI00129250	LRG1 Leucine-rich -α-2-glycoprotein	14	5.86	23	1.9	23	1.9	6	5.739	11	8.876	6	2.145	9	1.773	1.98	neovascularization
IPI00118130	ORM1 α-1-acid glycoprotein 1	9	4.37	14	2.31	14	2.3	19	3.452	27	37.976	8	2.616	38	3.206	2.70	acute-phase reaction

(a) Mouse IPI accession numbers are listed for each protein. IPI: International Protein Index. (b) No. of pep, the numbers of the matched the peptides with 95% confidence. (c) CRE/GFP ratio, Pre/On ratio and C/N ratio indicate the Cancer/Control ratio for each protein, C = cancer, N = normal. (d) Geometric standard deviation for the fold-change ratio. (e) Functional annotation from STRING.
