# Author Correction: Identification of urine protein biomarkers with the potential for early detection of lung cancer

**DOI:** 10.1038/s41598-018-32183-x

**Published:** 2018-09-28

**Authors:** Hongjuan Zhang, Jing Cao, Lin Li, Yanbin Liu, Hong Zhao, Nan Li, Bo Li, Aiqun Zhang, Huanwei Huang, She Chen, Mengqiu Dong, Lei Yu, Jian Zhang, Liang Chen

**Affiliations:** 1 School of Life Science, Tsinghua University, Beijing, 100084 China; 2 National Institute of Biological Sciences, Beijing, 102206 China; 3 Xijing Hospital, Xi’an, 510060 China; 4 General Hospital of the People’s Liberation Army, Beijing, 100853 China; 5 Beijing Tongren Hospital, Capital Medical University, Beijing, 100730 China; 6 National Institute of Biological Sciences, Collaborative Innovation Center for Cancer Medicine, Beijing, 102206 China

Correction to: *Scientific Reports* 10.1038/srep11805, published online 02 July 2015

The Acknowledgements section in this Article was omitted. The Acknowledgements section should read:

“This work was funded by NSFC (81472606) to L.C.”

