# Supplementary material for: Identification of urine protein biomarkers with the potential for early detection of lung cancer
Source: Sci Rep. 2015 Jul 2;5:11805. doi: 10.1038/srep11805 (PMC4488871; doi:10.1038/srep11805)
Supplement: Supplementary Information [file srep11805-s1.pdf]

Identification of urine protein biomarkers with the potential for early detection of lung cancer

Hongjuan Zhang[1*], Jing Cao[2*], Lin Li[1*], Yanbin Liu[1*], Hongzhao[1], Aiqun Zhang[3], Huanwei Huang[1], She Chen[1], Mengqiu Dong[1], Lei Yu[4], Jian Zhang[2#], Liang Chen[1,5#]

Running title: proteomic analysis of urine samples of Kras mutation positive mice and patients for efficient identification of biomarkers

LSL-KRAS^{G12D} and TetO- KRAS^{G12D} mouse model:

The LSL-KRAS^{G12D} knock-in mice kindly gifted by Tyler Jacks and crossed to FVB background, tumor induction was done by nasal inhalation of 10ul Adneo-Cre virus (Sino gene, 2.5*10^11pfu/ml) in 125ul DMEM(Hyclone,SH30022.01B) plus 0.6ul 2M Cacl2. 3.5-4 months after induction, panting mice of hunched posture were used for urine collection. The transgenic TetO- KRAS^{G12D} mouse was established in our laboratory, PCYL50 construct with TRE elements was used to drive KRAS^{G12D} expression, supplementary figure 1A shows the detailed map of this construct. Conventional pronuclei microinjection was conducted by transgenic center in our institute, founder #98 was confirmed for stable KRAS^{G12D} expression when crossed with CC10rtTA, and fed with doxycyclin diet.

Protein lysate preparation and trypsin digestion

The paired urine samples for quantitative mass spectrometric analysis were precipitated overnight by adding ten volumes of pre-chilled (-20°C) TCA:acetone(8:1), followed by centrifugation at 16,000 g for 20 minutes at 4°C. The protein pellet was washed with acetone once and dried in air briefly, then re-suspended with 2 M urea in 200 mM triethylammoniumbicarbonate(TAEB). Around 100 µg of protein from each sample was reduced by adding 1 µL of 200 mM TCEP and incubated at 55°C for 30 min, followed by alkylation by adding 1 µL of 375 mM iodoacetamide and incubated for 30 minutes in the dark. Around 2.5 µg of sequencing grade modified trypsin (Promega) was added to each protein solution and incubated at 37 °C overnight for trypsin digestion.

TMT Isobaric Reagent labeling

The obtained tryptic peptides were labeled individually with TMT2-126 for control, TMT2-127 for tumor according to the manufacturer's protocol, Label reagent was from TMTtwoplex Isobaric Labeling Reagent Set (Thermo, 90061, US). Briefly, each tube of 0.8 mg TMT Label Reagent was dissolved in 41 µL anhydrous acetonitrile and the solution was transferred to each sample of the digested tryptic peptides. The mixture was incubated at RT for 1 hr, and the reaction was quenched by adding 8 µL of 5% hydroxylamine. Equal amount of TMT labeled peptides from the two samples were mixed for the following SCX fractionation.

Fractionation of tryptic peptides by SCX chromatography

For TMT Reagent labeled peptides, around 50 µg of peptides were desalted and loaded onto a

home-made capillary SCX column (75 μm ID, 8 cm long) packed with 5 μm, 100 Å pore size Luna spherical strong cation exchange media (Phenomenex). The peptide mixture was fractionated with the gradient as shown in Table 1 generated by an Aglient 1200 quaternary pump system with 1μL/min flow rate (Buffer A: 7 mM KH_2PO_4, pH 2.65, 10% acetonitrile ; Buffer B: 7 mM KH_2PO_4, 350 mM KCl, pH 2.65, 10% acetonitrile; Buffer C: 50 mM K_2HPO_4, pH 7.5).

Supplementary Tables 1. SCX gradient for TMT Reagent labeled peptides.

Time (min)	Buffer A (%)	Buffer B (%)	Buffer C (%)
0	100	0	0
5	95	5	0
30	50	50	0
60	0	100	0
60.5	0	0	100
70	0	0	100

LC-MS/MS analyzes of fractionated peptides

Each fraction of peptides from TMT Reagent labeled peptides were separated by a home-made analytical capillary column (50 μm × 15 cm) packed with 5 μm spherical C18 reversed phase material (YMC). A Waters nanoAcquity UPLC system was used to generate the following HPLC gradient: 0-30% B in 75 min, 30-70% B in 15 min , 70-90% B in 5 min (A = 0.1% formic acid in water, B = 0.1% formic acid in acetonitrile). The eluted peptides were sprayed into a LTQ Orbitrap Velos mass spectrometer (Thermo) equipped with a nano-ESI ion source. The mass spectrometer was operated in data-dependent mode with one MS scan followed by ten HCD (High-energy Collisional Dissociation) scans for each cycle. Database searches were performed on an in-house Mascot server (Matrix Science Ltd.) against IPI (International Protein Index) mouse protein database. The search parameters were set as follows: 7 ppm mass tolerance for precursor ions; 0.02 Da mass tolerance for product ions; three missed cleavage sites were allowed for trypsin digestion; enzyme was chosen as semi-Trypsin which allows non-specific cleavage at peptide N-terminus; three variable modifications were icluded: protein N-Terminal acetylation, methionine oxidation, cysteine carbamidomethylation. For TMT Reagent labeled samples, two fixed modification were included: protein N-Terminal TMT labeling, lysine TMT labeling. The

search results were filtered with both peptide significance threshold and expectation value to be 0.05. The search results were further processed by Mascot Percolator to control the false-positive rate to be below 1%. The quantification of the two TMT reagent labeled samples was automatically done by Mascot search engine. The report ion mass tolerance was set to 10 ppm and only the unique peptides were used for quantification. The fold change ratio (CRE/GFP) < 0.67 (down-regulated) or > 1.5 (up-regulated) was defined as the cutoff value to designate significant changes in protein level.

Informatics analysis

To compare the overlapped protein in mice and human urine samples using IPI accession number, we firstly converted the IPI accession numbers of mice and human proteins to Ensemble Gene ID through DAVID (http://david.abcc.ncifcrf.gov/home.jsp), and the human Ensemble Gene IDs were then used to search for their mouse homologue genes through ensemble biomart （http://asia.ensembl.org/biomart/martview/）, then we compared the human to mouse homologue genes with mouse Ensemble Gene IDs to get overlapping proteins. Venny (http://bioinfogp.cnb.csic.es/tools/venny/) was utilized to compare different groups of urinary proteins and get the intersection charts. DAVID (http://david.abcc.ncifcrf.gov/home.jsp) was used to annotate cellular component and biological process for the differentially expressed proteins. Protein-protein interaction of the 7 up-regulated proteins was analyzed by STRING (Search Tool for the Retrieval of Interacting Genes/Proteins, version 9.1, http://string-db.org/).

Western blotting

Urine protein was precipitated by TCA:acetone and dissolved in 8M urea, due to the absence of a commonly recognized loading control in urine proteins, protein concentration was carefully quantified by Bradford protein assay kit (Bio-rad, 500-0006), and 20ug total urine proteins for each well were applied for western blotting . Proteins was separated by 8%w/v SDS-PAGE gel and transferred onto PVDF membrane (0.22um) using the wet transfer system (Bio-rad, 170-3940). The membrane was blocked with 5% milk in TBST for 1h at room temperature. Subsequently, membranes were incubated with rabbit anti mouse and human CP with 1:1000 dilution overnight at 4℃.After 4 times washing with TBST (0.1% Tween 20), the membranes were then incubated with 1:5000 dilution of goat anti-rabbit secondary antibody (Sigma, A6154) for 1h at room temperature. Then the membranes were washed 4 times with TBST (0.1% Tween 20) and colored

with ECL kit (Beijing PreGene Biotechnology Company, PE-500). Band intensity was measured by ImageJ.

ELISA Analysis

To quantify Orm1 protein level in human urine using Human alpha 1-Acid Glycoprotein Quantikine ELISA Kit (R&D, DAGP00), we firstly used ORM1 stock (200ng/ml) to produce a dilution series of standard solutions: 200ng/ml, 100ng/ml, 50ng/ml, 25ng/ml, 12.5ng/ml, 6.25ng/ml, 3.13ng/ml. Then we diluted urine samples from lung cancer patients and healthy control subjects by adding 20ul of urine to 180ul of dilution buffer supplied by the kit. Followed by the manufacturer's instructions, we added 100ul of assay diluents buffer to each well that had been pre-coated with a monoclonal antibody specific for human Orm1. Then we added 50ul of standard or sample per well, each experimental group was performed in two biological replicates and incubated for 2 hours at 37℃. After incubation, we discarded the supernatant and washed the plates four times with wash buffer. Then we added 200ul of HRP-conjugated polyclonal antibody against human Orm1 to each well and incubated 2 hours at 37℃. We then repeated the wash step as on above. Then we added 200ul of substrate solution and incubated 30 minutes in dark at room temperature, when the color changed from blue to yellow, we added 50ul of stop solution to each well and determined the optical density at 450nm. The protein concentration was determined with built-in standard curves side-by-side.

Supplementary Figure Legends

S1A. Schemetic map of pCYL50-TetO-Kras G12D for generating transgenic mice.

S1B. H&E staining of TetO- KRAS^{G12D} lung tissue before (Pre-Dox, upper panel) and 20 days after (On-Dox, lower panel) doxycycline treat (scale bars represent 200 μm and 50 μm in X40 and X200 magnification, respectively)..

S1C. Validation of rabbit anti mouse CP antibodies on 293T cells expressing mouse CP, wild-type 293T was used as negative control, 1:1000 dilution factor was applied.

S1D. Validation of rabbit anti human CP antibodies on 293T cells expressing human CP, wild-type 293T was used as negative control, 1:1000 dilution factor was applied.

S1E. Western blot analysis of CP in serum of 7 KRAS mutation cancer patients and 7 control subjects.

S1F. CP protein level in serum was analyzed by western blot on 36 cancer patients and 18 healthy controls. Band intensities were quantified by ImageJ, p=0.063.

Supplementary Figure 1

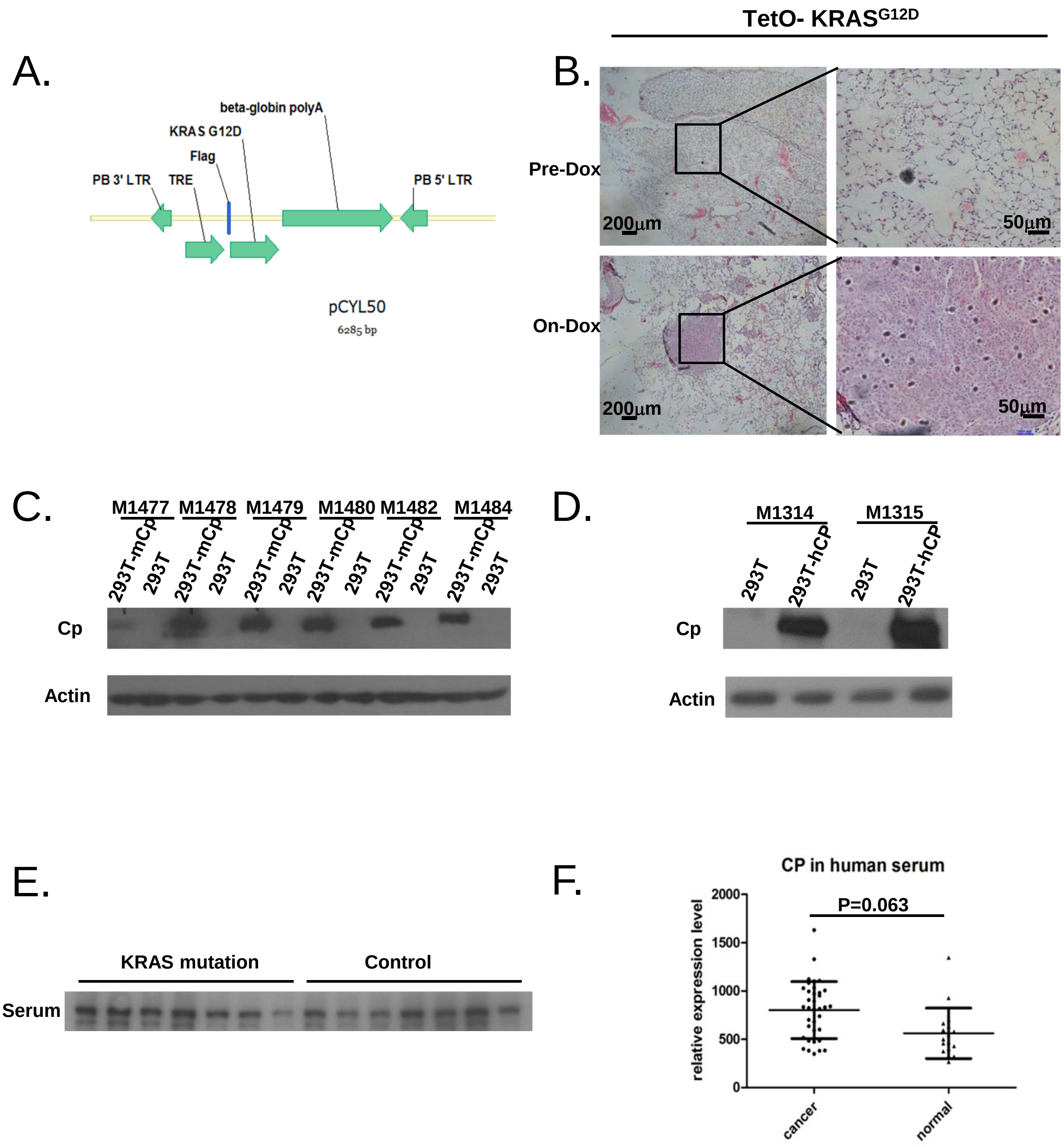

Supporting Information Tables 1

Biological processes of 346 proteins in mice urine

Category	Term	Term	Count	%	PValue	List Tota	Pop Hits	Pop Total	Fold Enri	Bonferror	Benjamini	FDR
GOTERM_BF	GO:000961	response	32	9.411765	1.95E-13	248	347	13588	5.05271	3.09E-10	3.09E-10	3.26E-10
GOTERM_BF	GO:000650	proteolys	41	12.05882	4.06E-06	248	1034	13588	2.172537	0.006425	4.96E-04	0.006798
GOTERM_BF	GO:000715	cell adhe	34	10	2.39E-09	248	561	13588	3.320626	3.79E-06	9.48E-07	4.00E-06
GOTERM_BF	GO:002261	biologica	34	10	2.50E-09	248	562	13588	3.314717	3.97E-06	7.93E-07	4.18E-06
GOTERM_BF	GO:000695	defense r	31	9.117647	6.67E-10	248	448	13588	3.791295	1.06E-06	3.52E-07	1.12E-06
GOTERM_BF	GO:000695	immune re	23	6.764706	4.99E-05	248	471	13588	2.675536	0.076119	0.003592	0.083467
GOTERM_BF	GO:000695	inflammat	21	6.176471	5.00E-09	248	225	13588	5.113763	7.93E-06	1.32E-06	8.36E-06
GOTERM_BF	GO:000681	ion trans	20	5.882353	0.060491	248	712	13588	1.539054	1	0.497037	64.78673
GOTERM_BF	GO:004259	homeostat	19	5.588235	0.020096	248	584	13588	1.782562	1	0.268453	28.79313
GOTERM_BF	GO:001003	response	18	5.294118	0.01097	248	505	13588	1.952922	1	0.180282	16.84857
GOTERM_BF	GO:000609	generatio	17	5	2.31E-05	248	261	13588	3.568718	0.036013	0.002036	0.038675
GOTERM_BF	GO:001972	cellular	16	4.705882	0.00154	248	343	13588	2.555817	0.913259	0.049659	2.545581
GOTERM_BF	GO:000681	cation tr	16	4.705882	0.049369	248	515	13588	1.702224	1	0.453243	57.12597
GOTERM_BF	GO:004298	regulatio	16	4.705882	0.079959	248	553	13588	1.585253	1	0.571411	75.19209
GOTERM_BF	GO:004306	regulatio	16	4.705882	0.086878	248	560	13588	1.565438	1	0.5938	78.13511
GOTERM_BF	GO:001094	regulatio	16	4.705882	0.090407	248	563	13588	1.557096	1	0.600034	79.50624
GOTERM_BF	GO:000252	acute inf	15	4.411765	1.84E-10	248	81	13588	10.14636	2.92E-07	1.46E-07	3.08E-07
GOTERM_BF	GO:001605	carbohydr	15	4.411765	1.84E-10	248	81	13588	10.14636	2.92E-07	1.46E-07	3.08E-07
GOTERM_BF	GO:001633	cell-cell	14	4.117647	3.86E-04	248	236	13588	3.250273	0.458079	0.017857	0.644058
GOTERM_BF	GO:000761	behavior	14	4.117647	0.03411	248	405	13588	1.893986	1	0.370322	44.04006
GOTERM_BF	GO:004206	wound hea	13	3.823529	8.82E-07	248	112	13588	6.359591	0.001398	1.55E-04	0.001476
GOTERM_BF	GO:000599	monosacch	13	3.823529	1.96E-04	248	191	13588	3.729184	0.266655	0.010285	0.326567
GOTERM_BF	GO:000268	positive	13	3.823529	3.90E-04	248	206	13588	3.457642	0.461656	0.017537	0.650998
GOTERM_BF	GO:004887	chemical	13	3.823529	0.035046	248	365	13588	1.951436	1	0.373497	44.93972
GOTERM_BF	GO:003210	regulatio	12	3.529412	2.62E-06	248	103	13588	6.383339	0.004146	3.78E-04	0.004382
GOTERM_BF	GO:001931	hexose me	12	3.529412	2.65E-04	248	169	13588	3.890437	0.342892	0.013454	0.441892
GOTERM_BF	GO:000687	cellular	12	3.529412	0.008316	248	261	13588	2.519095	0.999998	0.145876	13.03759
GOTERM_BF	GO:005508	cellular	12	3.529412	0.010012	248	268	13588	2.453298	1	0.169375	15.492
GOTERM_BF	GO:005080	ion homeo	12	3.529412	0.018474	248	293	13588	2.243972	1	0.258241	26.79589
GOTERM_BF	GO:005081	coagulati	11	3.235294	5.00E-07	248	70	13588	8.609908	7.93E-04	1.13E-04	8.37E-04
GOTERM_BF	GO:000759	blood coa	11	3.235294	5.00E-07	248	70	13588	8.609908	7.93E-04	1.13E-04	8.37E-04
GOTERM_BF	GO:000759	hemostasi	11	3.235294	5.73E-07	248	71	13588	8.488642	9.09E-04	1.14E-04	9.59E-04
GOTERM_BF	GO:005087	regulatio	11	3.235294	4.74E-06	248	89	13588	6.771838	0.007491	5.37E-04	0.00793

GOTERM_BF	GO:000597	polysacch	11	3.235294	6.41E-06	248	92	13588	6.551017	0.010114	6.77E-04	0.010721
GOTERM_BF	GO:001604	lipid cat	11	3.235294	1.67E-04	248	134	13588	4.497713	0.232169	0.009068	0.278247
GOTERM_BF	GO:00300(	cellular	11	3.235294	4.61E-04	248	152	13588	3.965089	0.518599	0.019564	0.768072
GOTERM_BF	GO:005508	cation hc	11	3.235294	0.001984	248	184	13588	3.275508	0.957104	0.061037	3.266645
GOTERM_BF	GO:004858	positive	11	3.235294	0.002148	248	186	13588	3.240288	0.966952	0.064672	3.532366
GOTERM_BF	GO:001567	monovaler	11	3.235294	0.050494	248	303	13588	1.989088	1	0.453509	57.96655
GOTERM_BF	GO:00650(	macromole	11	3.235294	0.089846	248	338	13588	1.783117	1	0.599883	79.29376
GOTERM_BF	GO:000697	response	10	2.941176	2.80E-05	248	87	13588	6.297738	0.043387	0.002332	0.046772
GOTERM_BF	GO:000225	immune ef	10	2.941176	4.86E-04	248	126	13588	4.348438	0.537752	0.020102	0.810554
GOTERM_BF	GO:000999	response	10	2.941176	7.20E-04	248	133	13588	4.119573	0.681121	0.026846	1.198215
GOTERM_BF	GO:005118	cofactor	10	2.941176	0.006131	248	182	13588	3.010457	0.999942	0.118975	9.775551
GOTERM_BF	GO:00430(	negative	10	2.941176	0.030762	248	239	13588	2.292482	1	0.345275	40.7049
GOTERM_BF	GO:00430(	negative	10	2.941176	0.034534	248	244	13588	2.245505	1	0.371546	44.44932
GOTERM_BF	GO:006054	negative	10	2.941176	0.035271	248	245	13588	2.23634	1	0.372994	45.154
GOTERM_BF	GO:00017(	in utero	10	2.941176	0.055695	248	267	13588	2.052072	1	0.472736	61.65647
GOTERM_BF	GO:000715	homophili	9	2.647059	0.001322	248	117	13588	4.21464	0.877226	0.044572	2.187827
GOTERM_BF	GO:00300(	cellular	9	2.647059	0.003105	248	134	13588	3.679947	0.992792	0.084314	5.06936
GOTERM_BF	GO:005077	positive	9	2.647059	0.003401	248	136	13588	3.62583	0.995495	0.083451	5.538805
GOTERM_BF	GO:00060(	glucose m	9	2.647059	0.004057	248	140	13588	3.522235	0.998414	0.091737	6.573364
GOTERM_BF	GO:000673	coenzyme	9	2.647059	0.004609	248	143	13588	3.448342	0.999343	0.100752	7.437178
GOTERM_BF	GO:005506	di-, tri-	9	2.647059	0.005219	248	146	13588	3.377486	0.999751	0.107452	8.380046
GOTERM_BF	GO:000646	protein c	9	2.647059	0.055648	248	227	13588	2.172304	1	0.474826	61.62408
GOTERM_BF	GO:007027	protein c	9	2.647059	0.055648	248	227	13588	2.172304	1	0.474826	61.62408
GOTERM_BF	GO:000602	aminoglyc	8	2.352941	4.01E-05	248	52	13588	8.42928	0.061652	0.003177	0.067092
GOTERM_BF	GO:000695	humoral i	8	2.352941	5.15E-05	248	54	13588	8.117085	0.07843	0.003545	0.086106
GOTERM_BF	GO:004427	cellular	8	2.352941	1.02E-04	248	60	13588	7.305376	0.149875	0.006226	0.171106
GOTERM_BF	GO:004603	ATP metak	8	2.352941	0.001249	248	90	13588	4.870251	0.862281	0.0431	2.06926
GOTERM_BF	GO:001003	response	8	2.352941	0.001421	248	92	13588	4.764376	0.895103	0.046841	2.350035
GOTERM_BF	GO:00516(	protein m	8	2.352941	0.001818	248	96	13588	4.56586	0.944221	0.057204	2.998343
GOTERM_BF	GO:005124	negative	8	2.352941	0.002297	248	100	13588	4.383226	0.973946	0.067742	3.77402
GOTERM_BF	GO:00092(	purine ri	8	2.352941	0.002431	248	101	13588	4.339828	0.978935	0.070244	3.989478
GOTERM_BF	GO:000919	ribonucle	8	2.352941	0.002571	248	102	13588	4.29728	0.983128	0.072808	4.213984
GOTERM_BF	GO:000914	purine nu	8	2.352941	0.003192	248	106	13588	4.135119	0.993717	0.0837	5.206699
GOTERM_BF	GO:004508	innate in	8	2.352941	0.003363	248	107	13588	4.096473	0.995219	0.083867	5.479579
GOTERM_BF	GO:003166	response	8	2.352941	0.005004	248	115	13588	3.811501	0.999649	0.104607	8.048212
GOTERM_BF	GO:000914	nucleosic	8	2.352941	0.005004	248	115	13588	3.811501	0.999649	0.104607	8.048212

GOTERM_BF	GO:000915	purine ri	8	2.352941	0.00602	248	119	13588	3.683383	0.999931	0.118393	9.607095
GOTERM_BF	GO:000925	ribonucle	8	2.352941	0.007824	248	125	13588	3.506581	0.999996	0.140948	12.31262
GOTERM_BF	GO:000616	purine nu	8	2.352941	0.026996	248	160	13588	2.739516	1	0.321275	36.73149
GOTERM_BF	GO:005124	positive	8	2.352941	0.029467	248	163	13588	2.689096	1	0.338007	39.36632
GOTERM_BF	GO:004405	regulatic	8	2.352941	0.074225	248	201	13588	2.180709	1	0.552792	72.47534
GOTERM_BF	GO:005081	regulatic	7	2.058824	1.06E-06	248	20	13588	19.17661	0.001684	1.69E-04	0.001778
GOTERM_BF	GO:000695	acute-pha	7	2.058824	1.40E-05	248	30	13588	12.78441	0.021929	0.001303	0.023383
GOTERM_BF	GO:000695	complemer	7	2.058824	4.19E-05	248	36	13588	10.65367	0.064235	0.003156	0.069998
GOTERM_BF	GO:000254	activatic	7	2.058824	4.19E-05	248	36	13588	10.65367	0.064235	0.003156	0.069998
GOTERM_BF	GO:000651	peptide m	7	2.058824	5.76E-05	248	38	13588	10.09295	0.08724	0.003796	0.096228
GOTERM_BF	GO:000609	glycolysi	7	2.058824	1.34E-04	248	44	13588	8.716642	0.191923	0.007582	0.2245
GOTERM_BF	GO:000600	glucose c	7	2.058824	3.43E-04	248	52	13588	7.37562	0.419868	0.016872	0.572633
GOTERM_BF	GO:001932	hexose ca	7	2.058824	3.43E-04	248	52	13588	7.37562	0.419868	0.016872	0.572633
GOTERM_BF	GO:004636	monosacch	7	2.058824	4.22E-04	248	54	13588	7.102449	0.488397	0.018445	0.704369
GOTERM_BF	GO:004616	alcohol c	7	2.058824	0.001144	248	65	13588	5.900496	0.837357	0.040437	1.897299
GOTERM_BF	GO:005160	protein m	7	2.058824	0.001144	248	65	13588	5.900496	0.837357	0.040437	1.897299
GOTERM_BF	GO:000675	ATP biosy	7	2.058824	0.003758	248	82	13588	4.677223	0.997451	0.089091	6.104322
GOTERM_BF	GO:000225	activatic	7	2.058824	0.004753	248	86	13588	4.459677	0.999477	0.100958	7.6602
GOTERM_BF	GO:001648	protein p	7	2.058824	0.005929	248	90	13588	4.26147	0.99992	0.118167	9.468484
GOTERM_BF	GO:000920	ribonucle	7	2.058824	0.006941	248	93	13588	4.124003	0.999984	0.130493	10.99756
GOTERM_BF	GO:000920	purine ri	7	2.058824	0.006941	248	93	13588	4.124003	0.999984	0.130493	10.99756
GOTERM_BF	GO:000914	purine nu	7	2.058824	0.007304	248	94	13588	4.08013	0.999991	0.135271	11.5409
GOTERM_BF	GO:003015	regulatic	7	2.058824	0.007304	248	94	13588	4.08013	0.999991	0.135271	11.5409
GOTERM_BF	GO:000914	nucleosic	7	2.058824	0.007681	248	95	13588	4.037182	0.999995	0.140137	12.10123
GOTERM_BF	GO:004343	response	7	2.058824	0.007681	248	95	13588	4.037182	0.999995	0.140137	12.10123
GOTERM_BF	GO:000915	purine ri	7	2.058824	0.013362	248	107	13588	3.584414	1	0.208991	20.14948
GOTERM_BF	GO:000926	ribonucle	7	2.058824	0.015776	248	111	13588	3.455246	1	0.231042	23.35634
GOTERM_BF	GO:000616	purine nu	7	2.058824	0.039036	248	137	13588	2.799506	1	0.394202	48.62718
GOTERM_BF	GO:000961	response	7	2.058824	0.066992	248	157	13588	2.442881	1	0.524354	68.6485
GOTERM_BF	GO:000972	response	7	2.058824	0.080766	248	165	13588	2.324438	1	0.572896	75.55364
GOTERM_BF	GO:000916	nucleotic	7	2.058824	0.09792	248	174	13588	2.204208	1	0.621998	82.16104
GOTERM_BF	GO:003019	regulatic	6	1.764706	3.34E-06	248	14	13588	23.48157	0.005279	4.41E-04	0.005583
GOTERM_BF	GO:000703	vacuole c	6	1.764706	3.45E-04	248	34	13588	9.66888	0.421164	0.016431	0.574978
GOTERM_BF	GO:003020	glycosami	6	1.764706	7.46E-04	248	40	13588	8.218548	0.693785	0.027147	1.240434
GOTERM_BF	GO:004424	cellular	6	1.764706	0.003422	248	56	13588	5.870392	0.995649	0.082687	5.573337
GOTERM_BF	GO:000244	lymphocyt	6	1.764706	0.012366	248	76	13588	4.325552	1	0.198871	18.79026

GOTERM_BF	GO:000246	adaptive	6	1.764706	0.018403	248	84	13588	3.913594	1	0.259626	26.70653
GOTERM_BF	GO:000225	adaptive	6	1.764706	0.018403	248	84	13588	3.913594	1	0.259626	26.70653
GOTERM_BF	GO:000269	regulatio	6	1.764706	0.022042	248	88	13588	3.735704	1	0.288161	31.12192
GOTERM_BF	GO:000244	leukocyte	6	1.764706	0.02302	248	89	13588	3.69373	1	0.294222	32.26474
GOTERM_BF	GO:000679	sulfur me	6	1.764706	0.028328	248	94	13588	3.497255	1	0.33191	38.16441
GOTERM_BF	GO:000150	ossificat	6	1.764706	0.044036	248	106	13588	3.101339	1	0.425172	52.91972
GOTERM_BF	GO:004362	cellular	6	1.764706	0.047073	248	108	13588	3.043907	1	0.442204	55.36057
GOTERM_BF	GO:000301	circulato	6	1.764706	0.051858	248	111	13588	2.961639	1	0.457735	58.96522
GOTERM_BF	GO:000801	blood cir	6	1.764706	0.051858	248	111	13588	2.961639	1	0.457735	58.96522
GOTERM_BF	GO:006034	bone deve	6	1.764706	0.064088	248	118	13588	2.785949	1	0.515416	66.97559
GOTERM_BF	GO:000854	epidermis	6	1.764706	0.077802	248	125	13588	2.629935	1	0.563413	74.20126
GOTERM_BF	GO:000681	sodium io	6	1.764706	0.077802	248	125	13588	2.629935	1	0.563413	74.20126
GOTERM_BF	GO:001087	lipid loc	6	1.764706	0.084127	248	128	13588	2.568296	1	0.586092	77.00659
GOTERM_BF	GO:000682	anion tra	6	1.764706	0.08849	248	130	13588	2.528784	1	0.5963	78.77183
GOTERM_BF	GO:005105	positive	6	1.764706	0.092969	248	132	13588	2.490469	1	0.608567	80.45088
GOTERM_BF	GO:000739	ectoderm	6	1.764706	0.095252	248	133	13588	2.471744	1	0.615712	81.25771
GOTERM_BF	GO:005081	negative	5	1.470588	1.25E-05	248	9	13588	30.43907	0.019628	0.001238	0.020905
GOTERM_BF	GO:003014	sphingoli	5	1.470588	6.70E-05	248	13	13588	21.0732	0.100764	0.004239	0.111956
GOTERM_BF	GO:004646	membrane	5	1.470588	6.70E-05	248	13	13588	21.0732	0.100764	0.004239	0.111956
GOTERM_BF	GO:000027	polysacch	5	1.470588	5.00E-04	248	21	13588	13.04531	0.547527	0.020129	0.832909
GOTERM_BF	GO:000704	lysosome	5	1.470588	6.02E-04	248	22	13588	12.45235	0.615405	0.023606	1.002763
GOTERM_BF	GO:005088	neuromusc	5	1.470588	0.00322	248	34	13588	8.0574	0.993991	0.083034	5.251235
GOTERM_BF	GO:004580	positive	5	1.470588	0.00322	248	34	13588	8.0574	0.993991	0.083034	5.251235
GOTERM_BF	GO:000030	response	5	1.470588	0.003975	248	36	13588	7.609767	0.998195	0.091277	6.445535
GOTERM_BF	GO:001598	ATP synth	5	1.470588	0.004394	248	37	13588	7.404098	0.999074	0.097619	7.102038
GOTERM_BF	GO:001598	energy co	5	1.470588	0.004394	248	37	13588	7.404098	0.999074	0.097619	7.102038
GOTERM_BF	GO:005125	protein p	5	1.470588	0.005828	248	40	13588	6.84879	0.999906	0.117752	9.315059
GOTERM_BF	GO:001081	regulatio	5	1.470588	0.005828	248	40	13588	6.84879	0.999906	0.117752	9.315059
GOTERM_BF	GO:003422	ion trans	5	1.470588	0.00694	248	42	13588	6.522657	0.999984	0.132032	10.99596
GOTERM_BF	GO:001599	proton tr	5	1.470588	0.014585	248	52	13588	5.2683	1	0.221633	21.78915
GOTERM_BF	GO:003010	regulatio	5	1.470588	0.014585	248	52	13588	5.2683	1	0.221633	21.78915
GOTERM_BF	GO:000681	hydrogen	5	1.470588	0.015556	248	53	13588	5.168898	1	0.230293	23.06868
GOTERM_BF	GO:000281	regulatio	5	1.470588	0.01871	248	56	13588	4.891993	1	0.258854	27.08977
GOTERM_BF	GO:000611	oxidative	5	1.470588	0.01871	248	56	13588	4.891993	1	0.258854	27.08977
GOTERM_BF	GO:000282	regulatio	5	1.470588	0.01871	248	56	13588	4.891993	1	0.258854	27.08977
GOTERM_BF	GO:000701	actin fil	5	1.470588	0.01871	248	56	13588	4.891993	1	0.258854	27.08977

GOTERM_BF	GO:005072	regulatio	5	1.470588	0.019843	248	57	13588	4.806169	1	0.270018	28.48532
GOTERM_BF	GO:005090	neuromusc	5	1.470588	0.023493	248	60	13588	4.56586	1	0.294688	32.81133
GOTERM_BF	GO:000666	sphingoli	5	1.470588	0.026138	248	62	13588	4.418574	1	0.315063	35.79116
GOTERM_BF	GO:000166	response	5	1.470588	0.028954	248	64	13588	4.280494	1	0.335529	38.82769
GOTERM_BF	GO:000664	membrane	5	1.470588	0.028954	248	64	13588	4.280494	1	0.335529	38.82769
GOTERM_BF	GO:000821	regulatio	5	1.470588	0.030427	248	65	13588	4.21464	1	0.344577	40.36168
GOTERM_BF	GO:000270	regulatio	5	1.470588	0.030427	248	65	13588	4.21464	1	0.344577	40.36168
GOTERM_BF	GO:007048	response	5	1.470588	0.030427	248	65	13588	4.21464	1	0.344577	40.36168
GOTERM_BF	GO:000270	regulatio	5	1.470588	0.038448	248	70	13588	3.913594	1	0.391926	48.09901
GOTERM_BF	GO:000758	response	5	1.470588	0.049524	248	76	13588	3.604626	1	0.451828	57.24224
GOTERM_BF	GO:006024	anatomica	5	1.470588	0.064431	248	83	13588	3.300622	1	0.514938	67.17726
GOTERM_BF	GO:006062	regulatio	5	1.470588	0.064431	248	83	13588	3.300622	1	0.514938	67.17726
GOTERM_BF	GO:001988	antigen p	5	1.470588	0.073889	248	87	13588	3.148869	1	0.55347	72.30774
GOTERM_BF	GO:000691	anti-apop	5	1.470588	0.076358	248	88	13588	3.113087	1	0.558702	73.51708
GOTERM_BF	GO:003019	negative	4	1.176471	1.14E-04	248	6	13588	36.52688	0.165374	0.006673	0.190477
GOTERM_BF	GO:000695	complemer	4	1.176471	6.48E-04	248	10	13588	21.91613	0.642224	0.024758	1.078207
GOTERM_BF	GO:000190	cell kill	4	1.176471	0.002789	248	16	13588	13.69758	0.988079	0.077378	4.564294
GOTERM_BF	GO:000608	cellular	4	1.176471	0.003342	248	17	13588	12.89184	0.995051	0.084674	5.444975
GOTERM_BF	GO:000602	aminoglyc	4	1.176471	0.003956	248	18	13588	12.17563	0.99814	0.092198	6.416148
GOTERM_BF	GO:005076	positive	4	1.176471	0.008032	248	23	13588	9.528752	0.999997	0.142813	12.61944
GOTERM_BF	GO:003109	regenerat	4	1.176471	0.008032	248	23	13588	9.528752	0.999997	0.142813	12.61944
GOTERM_BF	GO:000674	glutathic	4	1.176471	0.009058	248	24	13588	9.13172	0.999999	0.156151	14.11894
GOTERM_BF	GO:004254	response	4	1.176471	0.009058	248	24	13588	9.13172	0.999999	0.156151	14.11894
GOTERM_BF	GO:005076	regulatio	4	1.176471	0.010157	248	25	13588	8.766452	1	0.169817	15.69847
GOTERM_BF	GO:001081	positive	4	1.176471	0.012578	248	27	13588	8.117085	1	0.199936	19.08225
GOTERM_BF	GO:000910	coenzyme	4	1.176471	0.015302	248	29	13588	7.557286	1	0.229082	22.73569
GOTERM_BF	GO:000687	cellular	4	1.176471	0.015302	248	29	13588	7.557286	1	0.229082	22.73569
GOTERM_BF	GO:005118	cofactor	4	1.176471	0.019962	248	32	13588	6.84879	1	0.269142	28.63037
GOTERM_BF	GO:003409	response	4	1.176471	0.019962	248	32	13588	6.84879	1	0.269142	28.63037
GOTERM_BF	GO:000282	positive	4	1.176471	0.023456	248	34	13588	6.44592	1	0.296593	32.76896
GOTERM_BF	GO:000282	positive	4	1.176471	0.023456	248	34	13588	6.44592	1	0.296593	32.76896
GOTERM_BF	GO:005507	iron ion	4	1.176471	0.025319	248	35	13588	6.261751	1	0.311438	34.88256
GOTERM_BF	GO:000270	positive	4	1.176471	0.035789	248	40	13588	5.479032	1	0.374961	45.64537
GOTERM_BF	GO:000270	positive	4	1.176471	0.035789	248	40	13588	5.479032	1	0.374961	45.64537
GOTERM_BF	GO:004578	positive	4	1.176471	0.042983	248	43	13588	5.096774	1	0.41979	52.04443
GOTERM_BF	GO:003166	cellular	4	1.176471	0.048148	248	45	13588	4.870251	1	0.447278	56.19542

GOTERM_BF	GO:00026	positive	4	1.176471	0.05084	248	46	13588	4.764376	1	0.453406	58.22225
GOTERM_BF	GO:00068	oxygen ar	4	1.176471	0.053604	248	47	13588	4.663006	1	0.464277	60.21107
GOTERM_BF	GO:00068	chloride	4	1.176471	0.099285	248	61	13588	3.592808	1	0.625184	82.60751
GOTERM_BF	GO:00464	glycosphi	3	0.882353	0.003174	248	5	13588	32.87419	0.99354	0.084658	5.178928
GOTERM_BF	GO:00427	fibrinoly	3	0.882353	0.003174	248	5	13588	32.87419	0.99354	0.084658	5.178928
GOTERM_BF	GO:00193	glycolipi	3	0.882353	0.004704	248	6	13588	27.39516	0.999435	0.101325	7.584322
GOTERM_BF	GO:00311	organ reg	3	0.882353	0.004704	248	6	13588	27.39516	0.999435	0.101325	7.584322
GOTERM_BF	GO:00068	cellular	3	0.882353	0.013451	248	10	13588	16.4371	1	0.208211	20.2705
GOTERM_BF	GO:00467	muscle ma	3	0.882353	0.016245	248	11	13588	14.94282	1	0.234936	23.96442
GOTERM_BF	GO:00550	copper ic	3	0.882353	0.016245	248	11	13588	14.94282	1	0.234936	23.96442
GOTERM_BF	GO:00199	lipid stc	3	0.882353	0.022496	248	13	13588	12.64392	1	0.290837	31.6541
GOTERM_BF	GO:00028	positive	3	0.882353	0.025934	248	14	13588	11.74078	1	0.31536	35.56672
GOTERM_BF	GO:00027	positive	3	0.882353	0.025934	248	14	13588	11.74078	1	0.31536	35.56672
GOTERM_BF	GO:00301	platelet	3	0.882353	0.033397	248	16	13588	10.27319	1	0.366532	43.34481
GOTERM_BF	GO:00026	regulatic	3	0.882353	0.041585	248	18	13588	9.13172	1	0.41165	50.85982
GOTERM_BF	GO:00346	cellular	3	0.882353	0.041585	248	18	13588	9.13172	1	0.41165	50.85982
GOTERM_BF	GO:00507	regulatic	3	0.882353	0.041585	248	18	13588	9.13172	1	0.41165	50.85982
GOTERM_BF	GO:00066	glycosphi	3	0.882353	0.045932	248	19	13588	8.651104	1	0.436537	54.45799
GOTERM_BF	GO:00068	superoxic	3	0.882353	0.045932	248	19	13588	8.651104	1	0.436537	54.45799
GOTERM_BF	GO:00060	gluconeog	3	0.882353	0.050438	248	20	13588	8.218548	1	0.455572	57.92509
GOTERM_BF	GO:00427	hydrogen	3	0.882353	0.050438	248	20	13588	8.218548	1	0.455572	57.92509
GOTERM_BF	GO:00093	oligosacc	3	0.882353	0.050438	248	20	13588	8.218548	1	0.455572	57.92509
GOTERM_BF	GO:00066	glycolipi	3	0.882353	0.059897	248	22	13588	7.471408	1	0.495929	64.41268
GOTERM_BF	GO:00165	negative	3	0.882353	0.059897	248	22	13588	7.471408	1	0.495929	64.41268
GOTERM_BF	GO:00468	regulatic	3	0.882353	0.059897	248	22	13588	7.471408	1	0.495929	64.41268
GOTERM_BF	GO:00345	cellular	3	0.882353	0.059897	248	22	13588	7.471408	1	0.495929	64.41268
GOTERM_BF	GO:00060	tricarbox	3	0.882353	0.064836	248	23	13588	7.146564	1	0.51482	67.41447
GOTERM_BF	GO:00303	choleste	3	0.882353	0.064836	248	23	13588	7.146564	1	0.51482	67.41447
GOTERM_BF	GO:00159	sterol tr	3	0.882353	0.064836	248	23	13588	7.146564	1	0.51482	67.41447
GOTERM_BF	GO:00463	acetyl-Cc	3	0.882353	0.069907	248	24	13588	6.84879	1	0.537636	70.2473
GOTERM_BF	GO:00193	hexose bi	3	0.882353	0.075103	248	25	13588	6.574839	1	0.554832	72.90862
GOTERM_BF	GO:00060	pyruvate	3	0.882353	0.075103	248	25	13588	6.574839	1	0.554832	72.90862
GOTERM_BF	GO:00090	aerobic r	3	0.882353	0.085844	248	27	13588	6.087814	1	0.59151	77.71729
GOTERM_BF	GO:00425	response	3	0.882353	0.097014	248	29	13588	5.667964	1	0.620595	81.85902
GOTERM_BF	GO:00093	oligosacc	2	0.588235	0.036026	248	2	13588	54.79032	1	0.374557	45.86843
GOTERM_BF	GO:00461	aldehyde	2	0.588235	0.036026	248	2	13588	54.79032	1	0.374557	45.86843

GOTERM_BF	GO:004274	regulatic	2	0.588235	0.053552	248	3	13588	36.52688	1	0.466343	60.17451
GOTERM_BF	GO:000668	gangliosi	2	0.588235	0.053552	248	3	13588	36.52688	1	0.466343	60.17451
GOTERM_BF	GO:002241	circadiar	2	0.588235	0.053552	248	3	13588	36.52688	1	0.466343	60.17451
GOTERM_BF	GO:004518	regulatic	2	0.588235	0.053552	248	3	13588	36.52688	1	0.466343	60.17451
GOTERM_BF	GO:004274	circadiar	2	0.588235	0.07076	248	4	13588	27.39516	1	0.539738	70.7004
GOTERM_BF	GO:004593	negative	2	0.588235	0.07076	248	4	13588	27.39516	1	0.539738	70.7004
GOTERM_BF	GO:001089	negative	2	0.588235	0.07076	248	4	13588	27.39516	1	0.539738	70.7004
GOTERM_BF	GO:000704	lysosomal	2	0.588235	0.087657	248	5	13588	21.91613	1	0.594937	78.44478
GOTERM_BF	GO:000668	sphingomy	2	0.588235	0.087657	248	5	13588	21.91613	1	0.594937	78.44478

Supporting Information Tables 2

Bilogical processes of 50 up-regulated proteins in mice urine

Category	Term	Count	%	PValue	List Tota	Pop Hits	Pop Total	Fold Enri	Bonferror	Benjamini	FDR
GOTERM_BF	GO:000961response	12	24.4898	5.42E-09	43	347	13588	10.92795	2.94E-06	2.94E-06	7.90E-06
GOTERM_BF	GO:000695defense r	8	16.32653	4.01E-04	43	448	13588	5.642857	0.195717	0.016615	0.582611
GOTERM_BF	GO:000650proteolys	8	16.32653	0.037522	43	1034	13588	2.444874	1	0.324175	42.71406
GOTERM_BF	GO:000252acute inf	7	14.28571	1.64E-07	43	81	13588	27.30864	8.93E-05	4.46E-05	2.39E-04
GOTERM_BF	GO:003210regulatic	7	14.28571	6.89E-07	43	103	13588	21.47573	3.74E-04	1.25E-04	0.001003
GOTERM_BF	GO:000695inflammat	7	14.28571	6.15E-05	43	225	13588	9.831111	0.032822	0.005547	0.089491
GOTERM_BF	GO:000681cation tr	7	14.28571	0.004734	43	515	13588	4.295146	0.923969	0.094348	6.678932
GOTERM_BF	GO:000715cell adhe	7	14.28571	0.007138	43	561	13588	3.942959	0.979548	0.114455	9.909027
GOTERM_BF	GO:002261biologica	7	14.28571	0.007198	43	562	13588	3.935943	0.980214	0.112079	9.989047
GOTERM_BF	GO:000681ion trans	7	14.28571	0.021251	43	712	13588	3.106742	0.999991	0.232859	26.8684
GOTERM_BF	GO:005124positive	6	12.2449	1.39E-04	43	163	13588	11.6319	0.072614	0.00751	0.202037
GOTERM_BF	GO:005081coagulati	5	10.20408	6.24E-05	43	70	13588	22.57143	0.033295	0.004826	0.090802
GOTERM_BF	GO:000759blood coa	5	10.20408	6.24E-05	43	70	13588	22.57143	0.033295	0.004826	0.090802
GOTERM_BF	GO:000759hemostasi	5	10.20408	6.59E-05	43	71	13588	22.25352	0.03517	0.004465	0.096006
GOTERM_BF	GO:005087regulatic	5	10.20408	1.59E-04	43	89	13588	17.75281	0.082798	0.007826	0.231597
GOTERM_BF	GO:004206wound hea	5	10.20408	3.85E-04	43	112	13588	14.10714	0.188497	0.017255	0.558772
GOTERM_BF	GO:004858positive	5	10.20408	0.002535	43	186	13588	8.494624	0.74797	0.082532	3.629884
GOTERM_BF	GO:001567monovaler	5	10.20408	0.013945	43	303	13588	5.214521	0.999512	0.177597	18.50043
GOTERM_BF	GO:003000metal ior	5	10.20408	0.046782	43	442	13588	3.574661	1	0.371598	50.23974
GOTERM_BF	GO:000695immune re	5	10.20408	0.056656	43	471	13588	3.354565	1	0.415373	57.24282
GOTERM_BF	GO:004298regulatic	5	10.20408	0.090192	43	553	13588	2.857143	1	0.524716	74.76482
GOTERM_BF	GO:004306regulatic	5	10.20408	0.093425	43	560	13588	2.821429	1	0.532724	76.04035
GOTERM_BF	GO:001094regulatic	5	10.20408	0.094828	43	563	13588	2.806394	1	0.533252	76.57488
GOTERM_BF	GO:003019regulatic	4	8.163265	9.76E-06	43	14	13588	90.28571	0.005287	0.001324	0.014219
GOTERM_BF	GO:005081regulatic	4	8.163265	3.02E-05	43	20	13588	63.2	0.016255	0.003272	0.043956
GOTERM_BF	GO:000695acute-pha	4	8.163265	1.05E-04	43	30	13588	42.13333	0.055524	0.006327	0.153135
GOTERM_BF	GO:000675ATP biosy	4	8.163265	0.002052	43	82	13588	15.41463	0.672281	0.076593	2.94854
GOTERM_BF	GO:004603ATP metab	4	8.163265	0.002676	43	90	13588	14.04444	0.766657	0.082041	3.82885
GOTERM_BF	GO:000920purine ri	4	8.163265	0.002937	43	93	13588	13.5914	0.797571	0.084919	4.194824
GOTERM_BF	GO:000920ribonucle	4	8.163265	0.002937	43	93	13588	13.5914	0.797571	0.084919	4.194824
GOTERM_BF	GO:000914purine nu	4	8.163265	0.003028	43	94	13588	13.44681	0.807291	0.083013	4.321224
GOTERM_BF	GO:000914nucleosic	4	8.163265	0.00312	43	95	13588	13.30526	0.816716	0.081337	4.449838
GOTERM_BF	GO:005124negative	4	8.163265	0.003606	43	100	13588	12.64	0.859361	0.089178	5.126322

GOTERM_BF	GO:000920	purine ri	4	8.163265	0.003708	43	101	13588	12.51485	0.866998	0.087621	5.268333
GOTERM_BF	GO:000919	ribonucle	4	8.163265	0.003813	43	102	13588	12.39216	0.874343	0.086236	5.41259
GOTERM_BF	GO:000914	purine nu	4	8.163265	0.004247	43	106	13588	11.92453	0.90086	0.091809	6.012142
GOTERM_BF	GO:000915	purine ri	4	8.163265	0.004361	43	107	13588	11.81308	0.906795	0.090553	6.16767
GOTERM_BF	GO:000926	ribonucle	4	8.163265	0.004832	43	111	13588	11.38739	0.927918	0.092812	6.812367
GOTERM_BF	GO:000914	nucleosic	4	8.163265	0.005332	43	115	13588	10.9913	0.945157	0.098494	7.493187
GOTERM_BF	GO:000915	purine ri	4	8.163265	0.005863	43	119	13588	10.62185	0.958961	0.100972	8.21002
GOTERM_BF	GO:000925	ribonucle	4	8.163265	0.006718	43	125	13588	10.112	0.974268	0.111362	9.352318
GOTERM_BF	GO:005077	positive	4	8.163265	0.008468	43	136	13588	9.294118	0.990125	0.126997	11.6517
GOTERM_BF	GO:000616	purine nu	4	8.163265	0.008639	43	137	13588	9.226277	0.991009	0.125947	11.87362
GOTERM_BF	GO:000616	purine nu	4	8.163265	0.013147	43	160	13588	7.9	0.999243	0.17652	17.53393
GOTERM_BF	GO:000916	nucleotic	4	8.163265	0.016439	43	174	13588	7.264368	0.999877	0.20149	21.45168
GOTERM_BF	GO:003440	nucleobas	4	8.163265	0.017717	43	179	13588	7.061453	0.999939	0.206343	22.92555
GOTERM_BF	GO:003465	nucleobas	4	8.163265	0.017717	43	179	13588	7.061453	0.999939	0.206343	22.92555
GOTERM_BF	GO:000268	positive	4	8.163265	0.02556	43	206	13588	6.135922	0.999999	0.268339	31.42129
GOTERM_BF	GO:004427	nitrogen	4	8.163265	0.066125	43	302	13588	4.18543	1	0.440348	63.08661
GOTERM_BF	GO:003109	regenerat	3	6.122449	0.002265	43	23	13588	41.21739	0.70802	0.078794	3.24872
GOTERM_BF	GO:000695	complemer	3	6.122449	0.005498	43	36	13588	26.33333	0.949886	0.098074	7.716707
GOTERM_BF	GO:000254	activatic	3	6.122449	0.005498	43	36	13588	26.33333	0.949886	0.098074	7.716707
GOTERM_BF	GO:000695	humoral i	3	6.122449	0.012057	43	54	13588	17.55556	0.998622	0.167204	16.19753
GOTERM_BF	GO:005072	regulatic	3	6.122449	0.013369	43	57	13588	16.63158	0.99933	0.174962	17.80424
GOTERM_BF	GO:005160	protein m	3	6.122449	0.017155	43	65	13588	14.58462	0.999917	0.204804	22.28084
GOTERM_BF	GO:000225	activatic	3	6.122449	0.028943	43	86	13588	11.02326	1	0.287746	34.80902
GOTERM_BF	GO:000269	regulatic	3	6.122449	0.030196	43	88	13588	10.77273	1	0.293095	36.0238
GOTERM_BF	GO:001648	protein p	3	6.122449	0.031471	43	90	13588	10.53333	1	0.293382	37.23765
GOTERM_BF	GO:005160	protein m	3	6.122449	0.035419	43	96	13588	9.875	1	0.318836	40.86381
GOTERM_BF	GO:004508	innate im	3	6.122449	0.043125	43	107	13588	8.859813	1	0.352877	47.38529
GOTERM_BF	GO:003166	response	3	6.122449	0.049087	43	115	13588	8.243478	1	0.380899	51.96399
GOTERM_BF	GO:000681	sodium ic	3	6.122449	0.056933	43	125	13588	7.584	1	0.411685	57.4254
GOTERM_BF	GO:000225	immune ef	3	6.122449	0.057741	43	126	13588	7.52381	1	0.411055	57.95339
GOTERM_BF	GO:000999	response	3	6.122449	0.063505	43	133	13588	7.12782	1	0.437083	61.5487
GOTERM_BF	GO:000681	potassium	3	6.122449	0.087403	43	160	13588	5.925	1	0.523481	73.61447
GOTERM_BF	GO:003019	negative	2	4.081633	0.018406	43	6	13588	105.3333	0.999958	0.209113	23.71027
GOTERM_BF	GO:003110	organ reg	2	4.081633	0.018406	43	6	13588	105.3333	0.999958	0.209113	23.71027
GOTERM_BF	GO:005081	negative	2	4.081633	0.027485	43	9	13588	70.22222	1	0.280347	33.36849
GOTERM_BF	GO:000695	complemer	2	4.081633	0.030493	43	10	13588	63.2	1	0.290485	36.30853

GOTERM_BF	GO:000292	regulatic	2	4.081633	0.036482	43	12	13588	52.66667	1	0.321641	41.80574
GOTERM_BF	GO:000289	positive	2	4.081633	0.042435	43	14	13588	45.14286	1	0.353396	46.82919
GOTERM_BF	GO:000271	positive	2	4.081633	0.042435	43	14	13588	45.14286	1	0.353396	46.82919
GOTERM_BF	GO:000267	regulatic	2	4.081633	0.054232	43	18	13588	35.11111	1	0.406676	55.61439
GOTERM_BF	GO:000704	lysosome	2	4.081633	0.065888	43	22	13588	28.72727	1	0.444267	62.95003
GOTERM_BF	GO:005076	positive	2	4.081633	0.06878	43	23	13588	27.47826	1	0.4486	64.58635
GOTERM_BF	GO:005076	regulatic	2	4.081633	0.074538	43	25	13588	25.28	1	0.471284	67.6457
GOTERM_BF	GO:000288	regulatic	2	4.081633	0.088781	43	30	13588	21.06667	1	0.524034	74.1887
GOTERM_BF	GO:000271	regulatic	2	4.081633	0.088781	43	30	13588	21.06667	1	0.524034	74.1887
GOTERM_BF	GO:000695	complemer	2	4.081633	0.088781	43	30	13588	21.06667	1	0.524034	74.1887

Supporting Information Tables 3

KEGG pathways of 346 proteins in mice urine

Category	Term	Count	%	PValue	List	Pop Hits	Pop Total	Fold Enri	Bonferror	Benjamini	FDR
KEGG_PATH	mmu04142:Lysosome	31	9.117647	5.55E-22	154	119	5738	9.706319	6.38E-20	6.38E-20	6.32E-19
KEGG_PATH	mmu04610:Complement and coagulation cascac	20	5.882353	3.23E-14	154	75	5738	9.935931	3.72E-12	1.86E-12	3.68E-11
KEGG_PATH	mmu00511:Other glycan degradation	7	2.058824	2.09E-06	154	16	5738	16.30114	2.41E-04	8.02E-05	0.002384
KEGG_PATH	mmu00531:Glycosaminoglycan degradation	7	2.058824	2.26E-05	154	23	5738	11.33992	0.002595	6.49E-04	0.025737
KEGG_PATH	mmu04614:Renin-angiotensin system	6	1.764706	8.17E-05	154	18	5738	12.41991	0.009352	0.001877	0.09302
KEGG_PATH	mmu04514:Cell adhesion molecules (CAMs)	14	4.117647	2.11E-04	154	154	5738	3.387249	0.02395	0.004032	0.23982
KEGG_PATH	mmu00010:Glycolysis / Gluconeogenesis	8	2.352941	0.002072	154	68	5738	4.383499	0.212189	0.033497	2.334647
KEGG_PATH	mmu04512:ECM-receptor interaction	8	2.352941	0.006367	154	83	5738	3.5913	0.520273	0.087728	7.017266
KEGG_PATH	mmu00480:Glutathione metabolism	6	1.764706	0.01197	154	52	5738	4.299201	0.749644	0.14262	12.81799
KEGG_PATH	mmu00600:Sphingolipid metabolism	5	1.470588	0.024799	154	42	5738	4.435683	0.944305	0.250828	24.87709
KEGG_PATH	mmu04520:Adherens junction	6	1.764706	0.051402	154	76	5738	2.941558	0.997686	0.424024	45.17898
KEGG_PATH	mmu00500:Starch and sucrose metabolism	4	1.176471	0.069989	154	36	5738	4.139971	0.999762	0.501104	56.24244
KEGG_PATH	mmu04510:Focal adhesion	10	2.941176	0.081439	154	198	5738	1.881805	0.999943	0.528318	62.00105

Supporting Information Tables 4

KEGG pathways of 50 up-regulated proteins in mice urine

Category	Term	Count	%	PValue	List Tota	Pop Hits	Pop Total	Fold Enri	Bonferror	Benjamini	FDR
KEGG_PATH	mmu04610:	7	14.28571	9.66E-08	20	75	5738	26.77733	1.64E-06	1.64E-06	6.99E-05
KEGG_PATH	mmu04514:	3	6.122449	0.090879	20	154	5738	5.588961	0.802045	0.555079	49.83527

Supporting Information Tables 5

Sub-cellular distributions of 346 proteins in mice urine

Category		Term	Count	%	PValue	List Tota	Pop Hits	Pop Total	Fold Enri	Bonferror	Benjamini	FDR
GOTERM_CC	GO:00160Z	membrane	147	43.23529	0.007336	256	7536	15227	1.160248	0.287301	0.036934	6.722686
GOTERM_CC	GO:00444Z	membrane	130	38.23529	0.066561	256	6965	15227	1.110188	0.957933	0.232058	47.8498
GOTERM_CC	GO:00444Z	extracell	64	18.82353	1.15E-26	256	774	15227	4.918282	5.31E-25	2.66E-25	1.09E-23
GOTERM_CC	GO:000561	extracell	56	16.47059	3.22E-29	256	511	15227	6.518408	1.48E-27	1.48E-27	3.05E-26
GOTERM_CC	GO:003198	vesicle	25	7.352941	7.03E-06	256	519	15227	2.865148	3.23E-04	8.09E-05	0.006648
GOTERM_CC	GO:000026	cell frac	21	6.176471	0.002544	256	596	15227	2.095788	0.110583	0.014542	2.379182
GOTERM_CC	GO:004517	apical pa	19	5.588235	1.23E-11	256	136	15227	8.309771	5.68E-10	1.89E-10	1.17E-08
GOTERM_CC	GO:004299	cell proj	18	5.294118	0.016877	256	575	15227	1.861997	0.542953	0.07531	14.86056
GOTERM_CC	GO:000998	cell surf	17	5	5.78E-05	256	305	15227	3.315305	0.002656	5.32E-04	0.054638
GOTERM_CC	GO:003101	extracell	15	4.411765	7.35E-04	256	309	15227	2.887401	0.033236	0.005618	0.692105
GOTERM_CC	GO:00444Z	extracell	8	2.352941	8.89E-04	256	92	15227	5.172215	0.040067	0.005825	0.83671
GOTERM_CC	GO:003435	plasma li	3	0.882353	0.074566	256	27	15227	6.608941	0.971693	0.239822	51.92652
GOTERM_CC	GO:003299	protein-l	3	0.882353	0.074566	256	27	15227	6.608941	0.971693	0.239822	51.92652
GOTERM_CC	GO:000557	fibrinoge	2	0.588235	0.049406	256	3	15227	39.65365	0.902777	0.190943	38.05411
GOTERM_CC	GO:003125	trailing	2	0.588235	0.080985	256	5	15227	23.79219	0.979449	0.242316	54.9877

Supporting Information Tables 6

Sub-cellular distributions of 50 up-regulated proteins in mice urine

Category	Term		Count	%	PValue	List Tota	Pop Hits	Pop Total	Fold Enri	Bonferror	Benjamini	FDR
GOTERM_CC	GO:000561	extracell	17	34.69388	8.12E-15	37	511	15223	13.68758	3.65E-13	3.65E-13	7.63E-12
GOTERM_CC	GO:000588	plasma me	15	30.61224	0.004529	37	2906	15223	2.123705	0.184771	0.0971	4.179441
GOTERM_CC	GO:004445	plasma me	9	18.36735	0.03391	37	1633	15223	2.267539	0.788268	0.403979	27.70653
GOTERM_CC	GO:001646	proton-tr	2	4.081633	0.094778	37	42	15223	19.59202	0.988677	0.673793	60.79792

Supporting Information Tables 7

Biological processes of 318 proteins in human urine

Category	Term	Count	%	PValue	List Tota	Pop Hits	Pop Total	Fold Enri	Bonferror	Benjamini	FDR
GOTERM_BF	GO:000715cell adhe	53	17.0418	3.14E-17	266	700	13528	3.850612	6.61E-14	6.61E-14	5.43E-14
GOTERM_BF	GO:002261biologica	53	17.0418	3.33E-17	266	701	13528	3.845119	7.02E-14	3.51E-14	5.77E-14
GOTERM_BF	GO:000650proteolys	43	13.82637	7.41E-06	266	1054	13528	2.074817	0.015476	4.73E-04	0.012815
GOTERM_BF	GO:000961response	42	13.50482	3.41E-14	266	530	13528	4.030189	7.17E-11	2.39E-11	5.90E-11
GOTERM_BF	GO:001003response	40	12.86174	7.08E-09	266	721	13528	2.821478	1.49E-05	1.49E-06	1.22E-05
GOTERM_BF	GO:000695immune re	35	11.25402	6.49E-07	266	690	13528	2.57971	0.001365	6.83E-05	0.001123
GOTERM_BF	GO:000695defense r	32	10.28939	1.31E-06	266	615	13528	2.646225	0.002759	1.10E-04	0.00227
GOTERM_BF	GO:004259homeostat	24	7.717042	0.022552	266	751	13528	1.625262	1	0.247319	32.59982
GOTERM_BF	GO:004298regulatic	24	7.717042	0.043993	266	804	13528	1.518124	1	0.36714	54.07412
GOTERM_BF	GO:004306regulatic	24	7.717042	0.04815	266	812	13528	1.503167	1	0.384555	57.40866
GOTERM_BF	GO:001094regulatic	24	7.717042	0.049719	266	815	13528	1.497634	1	0.390249	58.60696
GOTERM_BF	GO:000695inflammat	23	7.395498	4.36E-07	266	325	13528	3.599121	9.18E-04	5.10E-05	7.54E-04
GOTERM_BF	GO:004212regulatic	23	7.395498	0.059172	266	787	13528	1.486295	1	0.425023	65.17959
GOTERM_BF	GO:000999response	21	6.752412	1.22E-08	266	220	13528	4.854545	2.56E-05	2.33E-06	2.10E-05
GOTERM_BF	GO:000692cell moti	21	6.752412	0.001093	266	475	13528	2.248421	0.899909	0.024189	1.873423
GOTERM_BF	GO:004306extracell	20	6.430868	4.43E-10	266	163	13528	6.24014	9.32E-07	1.55E-07	7.66E-07
GOTERM_BF	GO:004206wound hea	19	6.109325	3.85E-08	266	191	13528	5.059088	8.11E-05	6.24E-06	6.66E-05
GOTERM_BF	GO:003166response	19	6.109325	6.19E-08	266	197	13528	4.905004	1.30E-04	9.31E-06	1.07E-04
GOTERM_BF	GO:001003response	19	6.109325	1.13E-07	266	205	13528	4.713589	2.39E-04	1.49E-05	1.96E-04
GOTERM_BF	GO:004858positive	19	6.109325	9.21E-07	266	236	13528	4.094431	0.001937	9.23E-05	0.001593
GOTERM_BF	GO:000268positive	19	6.109325	1.04E-06	266	238	13528	4.060024	0.002193	9.54E-05	0.001803
GOTERM_BF	GO:000971response	19	6.109325	0.001061	266	405	13528	2.385891	0.893012	0.023746	1.81968
GOTERM_BF	GO:000972response	18	5.787781	9.21E-04	266	367	13528	2.494356	0.856209	0.021555	1.580894
GOTERM_BF	GO:001972cellular	18	5.787781	0.010488	266	466	13528	1.964439	1	0.144695	16.66997
GOTERM_BF	GO:004887chemical	18	5.787781	0.023693	266	512	13528	1.787946	1	0.256883	33.94742
GOTERM_BF	GO:003019extracell	17	5.466238	1.70E-10	266	104	13528	8.313187	3.58E-07	8.95E-08	2.94E-07
GOTERM_BF	GO:001605carbohydr	17	5.466238	3.50E-10	266	109	13528	7.931848	7.37E-07	1.47E-07	6.06E-07
GOTERM_BF	GO:005087regulatic	17	5.466238	1.62E-08	266	141	13528	6.131712	3.40E-05	2.83E-06	2.79E-05
GOTERM_BF	GO:000599monosacch	17	5.466238	7.77E-06	266	222	13528	3.894466	0.01623	4.81E-04	0.013444
GOTERM_BF	GO:001633cell-cell	17	5.466238	1.10E-04	266	276	13528	3.132505	0.207298	0.00374	0.190696
GOTERM_BF	GO:005081coagulati	16	5.144695	1.23E-09	266	102	13528	7.977591	2.59E-06	3.70E-07	2.13E-06
GOTERM_BF	GO:000759blood coa	16	5.144695	1.23E-09	266	102	13528	7.977591	2.59E-06	3.70E-07	2.13E-06
GOTERM_BF	GO:000759hemostasi	16	5.144695	2.78E-09	266	108	13528	7.534392	5.85E-06	7.32E-07	4.81E-06

GOTERM_BF	GO:00321(	regulatic	16	5.144695	5.29E-07	266	159	13528	5.1177	0.001112	5.86E-05	9.14E-04
GOTERM_BF	GO:001931	hexose me	16	5.144695	5.67E-06	266	192	13528	4.238095	0.011864	3.85E-04	0.009806
GOTERM_BF	GO:00156	blood ves	16	5.144695	9.86E-05	266	245	13528	3.321283	0.187428	0.003453	0.17039
GOTERM_BF	GO:000194	vasculatu	16	5.144695	1.29E-04	266	251	13528	3.24189	0.237858	0.004302	0.222932
GOTERM_BF	GO:001064	positive	16	5.144695	0.002152	266	329	13528	2.473296	0.989268	0.040028	3.657276
GOTERM_BF	GO:00430(	negative	16	5.144695	0.00424	266	354	13528	2.298628	0.99987	0.069593	7.085801
GOTERM_BF	GO:00430(	negative	16	5.144695	0.004818	266	359	13528	2.266614	0.999962	0.077518	8.014136
GOTERM_BF	GO:006054	negative	16	5.144695	0.004962	266	360	13528	2.260317	0.999972	0.078546	8.243636
GOTERM_BF	GO:000962	response	16	5.144695	0.00605	266	368	13528	2.21118	0.999997	0.092906	9.963587
GOTERM_BF	GO:000828	cell prol	16	5.144695	0.025026	266	436	13528	1.866317	1	0.264064	35.49065
GOTERM_BF	GO:004232	regulatic	16	5.144695	0.041453	266	466	13528	1.746168	1	0.356721	51.91716
GOTERM_BF	GO:00322(	regulatic	16	5.144695	0.047342	266	474	13528	1.716697	1	0.382182	56.77885
GOTERM_BF	GO:005117	regulatic	16	5.144695	0.05487	266	485	13528	1.677761	1	0.412981	62.32081
GOTERM_BF	GO:001922	regulatic	16	5.144695	0.05487	266	485	13528	1.677761	1	0.412981	62.32081
GOTERM_BF	GO:004308	positive	16	5.144695	0.087127	266	520	13528	1.564835	1	0.528816	79.33385
GOTERM_BF	GO:000252	acute inf	15	4.823151	6.54E-09	266	98	13528	7.784257	1.38E-05	1.53E-06	1.13E-05
GOTERM_BF	GO:005077	positive	15	4.823151	9.61E-07	266	145	13528	5.261084	0.002022	9.20E-05	0.001663
GOTERM_BF	GO:00099(	positive	15	4.823151	0.002073	266	295	13528	2.585956	0.987316	0.038926	3.524914
GOTERM_BF	GO:00400(	regulatic	15	4.823151	0.007493	266	341	13528	2.237118	1	0.113024	12.19779
GOTERM_BF	GO:00100:	response	14	4.501608	1.45E-06	266	129	13528	5.51938	0.003054	1.18E-04	0.002513
GOTERM_BF	GO:000758	response	14	4.501608	3.66E-06	266	140	13528	5.085714	0.00767	2.75E-04	0.006326
GOTERM_BF	GO:00060(	glucose m	14	4.501608	9.73E-06	266	153	13528	4.653595	0.020279	5.54E-04	0.016833
GOTERM_BF	GO:004854	response	14	4.501608	1.06E-04	266	192	13528	3.708333	0.199229	0.003636	0.18239
GOTERM_BF	GO:00071(	enzyme li	14	4.501608	0.017379	266	342	13528	2.081871	1	0.208299	26.15693
GOTERM_BF	GO:000687	cellular	14	4.501608	0.03335	266	374	13528	1.903743	1	0.311897	44.38156
GOTERM_BF	GO:005508	cellular	14	4.501608	0.036843	266	380	13528	1.873684	1	0.331793	47.75704
GOTERM_BF	GO:00508(	ion homec	14	4.501608	0.059883	266	409	13528	1.740831	1	0.42621	65.63164
GOTERM_BF	GO:00022{	immune ef	13	4.180064	1.26E-05	266	134	13528	4.933902	0.02617	6.80E-04	0.021787
GOTERM_BF	GO:004508	innate in	13	4.180064	1.70E-05	266	138	13528	4.79089	0.035124	8.31E-04	0.029374
GOTERM_BF	GO:001087	lipid loc	13	4.180064	6.11E-05	266	157	13528	4.211101	0.120715	0.002471	0.105647
GOTERM_BF	GO:005124	negative	13	4.180064	9.31E-05	266	164	13528	4.031359	0.178038	0.003318	0.160966
GOTERM_BF	GO:000691	anti-apop	13	4.180064	7.58E-04	266	206	13528	3.209431	0.797396	0.019051	1.303212
GOTERM_BF	GO:004851	blood ves	13	4.180064	9.35E-04	266	211	13528	3.133378	0.860413	0.021641	1.604884
GOTERM_BF	GO:005124	positive	13	4.180064	0.003158	266	244	13528	2.709602	0.998717	0.055787	5.324267
GOTERM_BF	GO:00300(	cellular	13	4.180064	0.004374	266	254	13528	2.602925	0.999902	0.071157	7.301114
GOTERM_BF	GO:005508	cation hc	13	4.180064	0.010855	266	286	13528	2.311688	1	0.147468	17.20245

GOTERM_BF	GO:00301	regulatic	12	3.858521	7.94E-05	266	137	13528	4.45464	0.153871	0.002979	0.137192
GOTERM_BF	GO:00511	cofactor	12	3.858521	0.001614	266	195	13528	3.12967	0.966612	0.03216	2.754622
GOTERM_BF	GO:00516	localizat	12	3.858521	0.039537	266	307	13528	1.987901	1	0.345955	50.22805
GOTERM_BF	GO:00488	cell moti	12	3.858521	0.039537	266	307	13528	1.987901	1	0.345955	50.22805
GOTERM_BF	GO:00060	generatic	12	3.858521	0.044657	266	313	13528	1.949795	1	0.370192	54.62297
GOTERM_BF	GO:00082	negative	12	3.858521	0.098361	266	361	13528	1.690542	1	0.55794	83.3181
GOTERM_BF	GO:00516	protein m	11	3.536977	6.71E-06	266	86	13528	6.504983	0.014034	4.42E-04	0.011613
GOTERM_BF	GO:00022	activatic	11	3.536977	1.49E-05	266	94	13528	5.951368	0.030969	7.67E-04	0.025845
GOTERM_BF	GO:00075	aging	11	3.536977	5.89E-05	266	110	13528	5.085714	0.116653	0.002429	0.101865
GOTERM_BF	GO:00059	polysacch	11	3.536977	6.37E-05	266	111	13528	5.039897	0.125414	0.002525	0.110045
GOTERM_BF	GO:00164	protein p	11	3.536977	6.87E-05	266	112	13528	4.994898	0.13467	0.002675	0.118778
GOTERM_BF	GO:00516	protein m	11	3.536977	1.41E-04	266	122	13528	4.58548	0.256856	0.004488	0.243625
GOTERM_BF	GO:00068	lipid tra	11	3.536977	5.70E-04	266	145	13528	3.858128	0.698577	0.015454	0.980527
GOTERM_BF	GO:00300	cellular	11	3.536977	0.014102	266	227	13528	2.464443	1	0.178548	21.77969
GOTERM_BF	GO:00604	epitheliu	11	3.536977	0.014102	266	227	13528	2.464443	1	0.178548	21.77969
GOTERM_BF	GO:00550	di-, tri-	11	3.536977	0.019244	266	239	13528	2.340705	1	0.223138	28.54413
GOTERM_BF	GO:00164	cell migr	11	3.536977	0.04565	266	276	13528	2.026915	1	0.375373	55.43171
GOTERM_BF	GO:00015	skeletal	11	3.536977	0.095369	266	319	13528	1.753695	1	0.553032	82.33421
GOTERM_BF	GO:00069	complemer	10	3.215434	9.40E-08	266	42	13528	12.10884	1.98E-04	1.32E-05	1.63E-04
GOTERM_BF	GO:00025	activatic	10	3.215434	1.17E-07	266	43	13528	11.82724	2.46E-04	1.45E-05	2.02E-04
GOTERM_BF	GO:00060	aminoglyc	10	3.215434	4.55E-06	266	65	13528	7.824176	0.009524	3.30E-04	0.007862
GOTERM_BF	GO:00024	lymphocyt	10	3.215434	8.49E-06	266	70	13528	7.265306	0.017717	5.11E-04	0.014687
GOTERM_BF	GO:00024	adaptive	10	3.215434	1.87E-05	266	77	13528	6.604824	0.03859	8.94E-04	0.03233
GOTERM_BF	GO:00022	adaptive	10	3.215434	1.87E-05	266	77	13528	6.604824	0.03859	8.94E-04	0.03233
GOTERM_BF	GO:00069	humoral i	10	3.215434	2.31E-05	266	79	13528	6.437613	0.047366	0.001078	0.039863
GOTERM_BF	GO:00461	alcohol c	10	3.215434	2.82E-05	266	81	13528	6.27866	0.057718	0.001264	0.048836
GOTERM_BF	GO:00442	cellular	10	3.215434	4.16E-05	266	85	13528	5.983193	0.083916	0.001787	0.07199
GOTERM_BF	GO:00024	leukocyte	10	3.215434	4.57E-05	266	86	13528	5.913621	0.091754	0.001923	0.079045
GOTERM_BF	GO:00085	epidermis	10	3.215434	0.010381	266	184	13528	2.763975	1	0.144256	16.51326
GOTERM_BF	GO:00512	regulatic	10	3.215434	0.01386	266	193	13528	2.635085	1	0.176811	21.44752
GOTERM_BF	GO:00073	ectoderm	10	3.215434	0.016617	266	199	13528	2.555635	1	0.20237	25.16058
GOTERM_BF	GO:00099	negative	10	3.215434	0.030231	266	221	13528	2.301228	1	0.297494	41.19462
GOTERM_BF	GO:00510	positive	10	3.215434	0.031718	266	223	13528	2.280589	1	0.30429	42.73485
GOTERM_BF	GO:00071	transmemb	10	3.215434	0.032301	266	224	13528	2.270408	1	0.306285	43.32854
GOTERM_BF	GO:00106	negative	10	3.215434	0.0555	266	248	13528	2.050691	1	0.415255	62.7524
GOTERM_BF	GO:00106	regulatic	10	3.215434	0.056714	266	249	13528	2.042456	1	0.420873	63.57231

GOTERM_BF	GO:005109	positive	10	3.215434	0.096805	266	278	13528	1.829394	1	0.555957	82.81297
GOTERM_BF	GO:001606	immunoglo	9	2.893891	9.30E-06	266	54	13528	8.47619	0.019395	5.44E-04	0.016091
GOTERM_BF	GO:001972	B cell me	9	2.893891	1.23E-05	266	56	13528	8.173469	0.025519	6.80E-04	0.021238
GOTERM_BF	GO:000926	response	9	2.893891	2.16E-04	266	83	13528	5.51463	0.365745	0.006673	0.373405
GOTERM_BF	GO:003196	response	9	2.893891	2.55E-04	266	85	13528	5.384874	0.415591	0.007537	0.440385
GOTERM_BF	GO:000716	cell-matr	9	2.893891	3.50E-04	266	89	13528	5.142857	0.521497	0.009911	0.603809
GOTERM_BF	GO:003158	cell-subs	9	2.893891	6.71E-04	266	98	13528	4.670554	0.756441	0.017286	1.1538
GOTERM_BF	GO:000269	regulatic	9	2.893891	8.19E-04	266	101	13528	4.531825	0.821834	0.019859	1.407398
GOTERM_BF	GO:000152	angiogene	9	2.893891	0.008725	266	148	13528	3.092664	1	0.126843	14.06432
GOTERM_BF	GO:000673	coenzyme	9	2.893891	0.010545	266	153	13528	2.991597	1	0.144486	16.75297
GOTERM_BF	GO:000697	response	9	2.893891	0.015521	266	164	13528	2.790941	1	0.1925	23.70392
GOTERM_BF	GO:000269	regulatic	9	2.893891	0.016582	266	166	13528	2.757315	1	0.203142	25.11388
GOTERM_BF	GO:003033	regulatic	9	2.893891	0.01827	266	169	13528	2.708369	1	0.216605	27.307
GOTERM_BF	GO:005086	regulatic	9	2.893891	0.021987	266	175	13528	2.61551	1	0.24567	31.92313
GOTERM_BF	GO:005124	negative	9	2.893891	0.031025	266	187	13528	2.44767	1	0.301348	42.02162
GOTERM_BF	GO:005196	regulatic	9	2.893891	0.035469	266	192	13528	2.383929	1	0.324194	46.45325
GOTERM_BF	GO:004001	regulatic	9	2.893891	0.035469	266	192	13528	2.383929	1	0.324194	46.45325
GOTERM_BF	GO:000155	regulatic	9	2.893891	0.037395	266	194	13528	2.359352	1	0.334511	48.27228
GOTERM_BF	GO:004559	positive	9	2.893891	0.081517	266	229	13528	1.998752	1	0.514094	77.0235
GOTERM_BF	GO:000695	complemer	8	2.572347	1.10E-06	266	29	13528	14.02956	0.002321	9.68E-05	0.001909
GOTERM_BF	GO:000245	humoral i	8	2.572347	1.80E-06	266	31	13528	13.12442	0.003778	1.40E-04	0.00311
GOTERM_BF	GO:003019	regulatic	8	2.572347	5.25E-06	266	36	13528	11.30159	0.010992	3.68E-04	0.009081
GOTERM_BF	GO:005081	regulatic	8	2.572347	1.30E-05	266	41	13528	9.923345	0.027007	6.84E-04	0.022493
GOTERM_BF	GO:000609	pyruvate	8	2.572347	1.53E-05	266	42	13528	9.687075	0.031794	7.69E-04	0.026544
GOTERM_BF	GO:000609	glycolysi	8	2.572347	3.29E-05	266	47	13528	8.656535	0.066942	0.001442	0.056915
GOTERM_BF	GO:003020	glycosami	8	2.572347	9.29E-05	266	55	13528	7.397403	0.177705	0.003368	0.160634
GOTERM_BF	GO:000600	glucose c	8	2.572347	1.31E-04	266	58	13528	7.014778	0.241021	0.004234	0.226342
GOTERM_BF	GO:001932	hexose ca	8	2.572347	3.92E-04	266	69	13528	5.89648	0.561481	0.010931	0.675047
GOTERM_BF	GO:004636	monosacch	8	2.572347	4.67E-04	266	71	13528	5.730382	0.625814	0.012851	0.80444
GOTERM_BF	GO:005138	response	8	2.572347	8.26E-04	266	78	13528	5.216117	0.824346	0.019793	1.418899
GOTERM_BF	GO:001974	secondary	8	2.572347	8.91E-04	266	79	13528	5.15009	0.846962	0.021104	1.530476
GOTERM_BF	GO:000268	negative	8	2.572347	0.001195	266	83	13528	4.901893	0.919283	0.025613	2.046723
GOTERM_BF	GO:000715	homophili	8	2.572347	0.014611	266	131	13528	3.10578	1	0.183313	22.475
GOTERM_BF	GO:003288	regulatic	8	2.572347	0.018929	266	138	13528	2.94824	1	0.221095	28.1465
GOTERM_BF	GO:005112	negative	8	2.572347	0.021764	266	142	13528	2.865191	1	0.244758	31.65336
GOTERM_BF	GO:005124	regulatic	8	2.572347	0.026547	266	148	13528	2.749035	1	0.27253	37.20901

GOTERM_BF	GO:004648	glyceroli	8	2.572347	0.040378	266	162	13528	2.511464	1	0.350553	50.97586
GOTERM_BF	GO:001074	positive	8	2.572347	0.046277	266	167	13528	2.43627	1	0.376682	55.93555
GOTERM_BF	GO:000193	regulatio	8	2.572347	0.053987	266	173	13528	2.351775	1	0.411998	61.70722
GOTERM_BF	GO:001604	lipid cat	8	2.572347	0.053987	266	173	13528	2.351775	1	0.411998	61.70722
GOTERM_BF	GO:005134	positive	8	2.572347	0.062521	266	179	13528	2.272945	1	0.437772	67.26203
GOTERM_BF	GO:003226	negative	8	2.572347	0.064019	266	180	13528	2.260317	1	0.44298	68.15519
GOTERM_BF	GO:000801	blood cir	8	2.572347	0.073475	266	186	13528	2.187404	1	0.485113	73.28449
GOTERM_BF	GO:000301	circulato	8	2.572347	0.073475	266	186	13528	2.187404	1	0.485113	73.28449
GOTERM_BF	GO:000961	response	8	2.572347	0.085467	266	193	13528	2.108068	1	0.523083	78.67416
GOTERM_BF	GO:000940	response	7	2.250804	7.63E-04	266	56	13528	6.357143	0.799429	0.018944	1.311394
GOTERM_BF	GO:004360	cellular	7	2.250804	7.63E-04	266	56	13528	6.357143	0.799429	0.018944	1.311394
GOTERM_BF	GO:004576	regulatio	7	2.250804	0.001425	266	63	13528	5.650794	0.950265	0.029565	2.435682
GOTERM_BF	GO:003327	response	7	2.250804	0.001814	266	66	13528	5.393939	0.978137	0.035098	3.092333
GOTERM_BF	GO:003109	regenerat	7	2.250804	0.00228	266	69	13528	5.15942	0.991814	0.041634	3.871451
GOTERM_BF	GO:003463	cellular	7	2.250804	0.00228	266	69	13528	5.15942	0.991814	0.041634	3.871451
GOTERM_BF	GO:000666	sphingoli	7	2.250804	0.003475	266	75	13528	4.746667	0.999343	0.059725	5.843753
GOTERM_BF	GO:000676	vitamin m	7	2.250804	0.003475	266	75	13528	4.746667	0.999343	0.059725	5.843753
GOTERM_BF	GO:005072	regulatio	7	2.250804	0.003713	266	76	13528	4.684211	0.999602	0.06266	6.230856
GOTERM_BF	GO:000664	membrane	7	2.250804	0.005084	266	81	13528	4.395062	0.999978	0.079803	8.43785
GOTERM_BF	GO:004232	positive	7	2.250804	0.011966	266	97	13528	3.670103	1	0.160337	18.79582
GOTERM_BF	GO:001056	positive	7	2.250804	0.013757	266	100	13528	3.56	1	0.177738	21.30462
GOTERM_BF	GO:004593	positive	7	2.250804	0.013757	266	100	13528	3.56	1	0.177738	21.30462
GOTERM_BF	GO:004858	negative	7	2.250804	0.013757	266	100	13528	3.56	1	0.177738	21.30462
GOTERM_BF	GO:001605	carbohydr	7	2.250804	0.018647	266	107	13528	3.327103	1	0.219361	27.78807
GOTERM_BF	GO:000756	female pr	7	2.250804	0.021067	266	110	13528	3.236364	1	0.240399	30.8061
GOTERM_BF	GO:005122	regulatio	7	2.250804	0.024611	266	114	13528	3.122807	1	0.261554	35.01415
GOTERM_BF	GO:003284	regulatio	7	2.250804	0.024611	266	114	13528	3.122807	1	0.261554	35.01415
GOTERM_BF	GO:000679	sulfur me	7	2.250804	0.025556	266	115	13528	3.095652	1	0.267578	36.09428
GOTERM_BF	GO:005086	regulatio	7	2.250804	0.027517	266	117	13528	3.042735	1	0.279733	38.28289
GOTERM_BF	GO:007020	regulatio	7	2.250804	0.031733	266	121	13528	2.942149	1	0.303068	42.75036
GOTERM_BF	GO:000166	response	7	2.250804	0.048249	266	134	13528	2.656716	1	0.383793	57.48544
GOTERM_BF	GO:003085	epithelia	7	2.250804	0.052693	266	137	13528	2.59854	1	0.405662	60.79078
GOTERM_BF	GO:007048	response	7	2.250804	0.058993	266	141	13528	2.524823	1	0.426785	65.06472
GOTERM_BF	GO:004343	response	7	2.250804	0.082449	266	154	13528	2.311688	1	0.514039	77.4234
GOTERM_BF	GO:004362	cellular	7	2.250804	0.099123	266	162	13528	2.197531	1	0.559524	83.56004
GOTERM_BF	GO:003019	negative	6	1.92926	7.00E-05	266	23	13528	13.26708	0.137	0.002675	0.12099

GOTERM_BF	GO:005081	negative	6	1.92926	1.30E-04	266	26	13528	11.73626	0.240066	0.00428	0.225311
GOTERM_BF	GO:003019	collagen	6	1.92926	2.24E-04	266	29	13528	10.52217	0.376496	0.006823	0.3874
GOTERM_BF	GO:001931	hexose bi	6	1.92926	3.11E-04	266	31	13528	9.843318	0.480381	0.008928	0.536463
GOTERM_BF	GO:000687	cellular	6	1.92926	3.11E-04	266	31	13528	9.843318	0.480381	0.008928	0.536463
GOTERM_BF	GO:005507	iron ion	6	1.92926	6.37E-04	266	36	13528	8.47619	0.73851	0.016836	1.096088
GOTERM_BF	GO:004636	monosacch	6	1.92926	7.25E-04	266	37	13528	8.247104	0.782703	0.018444	1.246423
GOTERM_BF	GO:001591	sterol tr	6	1.92926	7.25E-04	266	37	13528	8.247104	0.782703	0.018444	1.246423
GOTERM_BF	GO:003030	cholester	6	1.92926	7.25E-04	266	37	13528	8.247104	0.782703	0.018444	1.246423
GOTERM_BF	GO:000676	nicotinam	6	1.92926	0.001043	266	40	13528	7.628571	0.888885	0.0236	1.789143
GOTERM_BF	GO:004649	nicotinam	6	1.92926	0.001043	266	40	13528	7.628571	0.888885	0.0236	1.789143
GOTERM_BF	GO:000982	alkaloid	6	1.92926	0.001169	266	41	13528	7.442509	0.914823	0.02533	2.003425
GOTERM_BF	GO:000667	ceramide	6	1.92926	0.001306	266	42	13528	7.265306	0.936197	0.027691	2.235795
GOTERM_BF	GO:001936	pyridine	6	1.92926	0.001306	266	42	13528	7.265306	0.936197	0.027691	2.235795
GOTERM_BF	GO:003318	response	6	1.92926	0.001306	266	42	13528	7.265306	0.936197	0.027691	2.235795
GOTERM_BF	GO:000716	negative	6	1.92926	0.001455	266	43	13528	7.096346	0.953355	0.029604	2.487088
GOTERM_BF	GO:004616	alcohol b	6	1.92926	0.001616	266	44	13528	6.935065	0.966758	0.031899	2.758127
GOTERM_BF	GO:004651	sphingoic	6	1.92926	0.001789	266	45	13528	6.780952	0.976935	0.034936	3.049726
GOTERM_BF	GO:001081	regulatic	6	1.92926	0.001976	266	46	13528	6.63354	0.984438	0.037812	3.362681
GOTERM_BF	GO:000673	oxidoredu	6	1.92926	0.003406	266	52	13528	5.868132	0.99924	0.059542	5.730774
GOTERM_BF	GO:003016	regulatic	6	1.92926	0.003702	266	53	13528	5.757412	0.999593	0.06299	6.213567
GOTERM_BF	GO:000651	peptide m	6	1.92926	0.003702	266	53	13528	5.757412	0.999593	0.06299	6.213567
GOTERM_BF	GO:004547	response	6	1.92926	0.00827	266	64	13528	4.767857	1	0.122303	13.37945
GOTERM_BF	GO:000698	response	6	1.92926	0.012653	266	71	13528	4.297787	1	0.166687	19.76778
GOTERM_BF	GO:000701	actin fil	6	1.92926	0.013386	266	72	13528	4.238095	1	0.174427	20.7918
GOTERM_BF	GO:005127	positive	6	1.92926	0.043377	266	98	13528	3.113703	1	0.364374	53.5596
GOTERM_BF	GO:006024	anatomica	6	1.92926	0.057234	266	106	13528	2.878706	1	0.421044	63.91766
GOTERM_BF	GO:000269	positive	6	1.92926	0.057234	266	106	13528	2.878706	1	0.421044	63.91766
GOTERM_BF	GO:005178	response	6	1.92926	0.059125	266	107	13528	2.851802	1	0.426138	65.1495
GOTERM_BF	GO:000165	urogenita	6	1.92926	0.065012	266	110	13528	2.774026	1	0.44681	68.73451
GOTERM_BF	GO:005086	positive	6	1.92926	0.067044	266	111	13528	2.749035	1	0.455929	69.88957
GOTERM_BF	GO:005160	detectior	6	1.92926	0.082251	266	118	13528	2.585956	1	0.514559	77.33907
GOTERM_BF	GO:001407	response	6	1.92926	0.089283	266	121	13528	2.521842	1	0.535141	80.16212
GOTERM_BF	GO:003019	positive	5	1.607717	2.75E-05	266	10	13528	25.42857	0.056341	0.00126	0.047637
GOTERM_BF	GO:005082	positive	5	1.607717	8.95E-05	266	13	13528	19.56044	0.171785	0.003301	0.154749
GOTERM_BF	GO:004646	membrane	5	1.607717	2.80E-04	266	17	13528	14.95798	0.445579	0.008159	0.483467
GOTERM_BF	GO:003014	sphingoli	5	1.607717	2.80E-04	266	17	13528	14.95798	0.445579	0.008159	0.483467

GOTERM_BF	GO:000602	aminoglyc	5	1.607717	6.62E-04	266	21	13528	12.10884	0.752114	0.017284	1.139497
GOTERM_BF	GO:004586	negative	5	1.607717	7.97E-04	266	22	13528	11.55844	0.813403	0.019557	1.369936
GOTERM_BF	GO:001652	negative	5	1.607717	9.50E-04	266	23	13528	11.0559	0.864849	0.021753	1.63099
GOTERM_BF	GO:005118	vitamin t	5	1.607717	0.001123	266	24	13528	10.59524	0.906069	0.024589	1.924619
GOTERM_BF	GO:000609	gluconeog	5	1.607717	0.001316	266	25	13528	10.17143	0.937528	0.027622	2.252728
GOTERM_BF	GO:005077	negative	5	1.607717	0.001316	266	25	13528	10.17143	0.937528	0.027622	2.252728
GOTERM_BF	GO:000668	glycosphi	5	1.607717	0.001532	266	26	13528	9.78022	0.960349	0.03085	2.617154
GOTERM_BF	GO:001081	positive	5	1.607717	0.002035	266	28	13528	9.081633	0.986267	0.038575	3.461893
GOTERM_BF	GO:000027	polysacch	5	1.607717	0.002035	266	28	13528	9.081633	0.986267	0.038575	3.461893
GOTERM_BF	GO:000666	glycolipi	5	1.607717	0.002987	266	31	13528	8.202765	0.998157	0.053281	5.042101
GOTERM_BF	GO:003252	response	5	1.607717	0.003767	266	33	13528	7.705628	0.999646	0.063049	6.319686
GOTERM_BF	GO:005159	response	5	1.607717	0.008985	266	42	13528	6.054422	1	0.129487	14.45266
GOTERM_BF	GO:000602	proteogly	5	1.607717	0.009757	266	43	13528	5.913621	1	0.138913	15.59838
GOTERM_BF	GO:00027(	regulatic	5	1.607717	0.021173	266	54	13528	4.708995	1	0.240189	30.93651
GOTERM_BF	GO:004508	regulatic	5	1.607717	0.021173	266	54	13528	4.708995	1	0.240189	30.93651
GOTERM_BF	GO:004254	response	5	1.607717	0.023862	266	56	13528	4.540816	1	0.257176	34.14481
GOTERM_BF	GO:000281	regulatic	5	1.607717	0.023862	266	56	13528	4.540816	1	0.257176	34.14481
GOTERM_BF	GO:004578	positive	5	1.607717	0.029834	266	60	13528	4.238095	1	0.295531	40.77709
GOTERM_BF	GO:00027(	regulatic	5	1.607717	0.031453	266	61	13528	4.168618	1	0.303493	42.4633
GOTERM_BF	GO:005127	negative	5	1.607717	0.034843	266	63	13528	4.036281	1	0.320775	45.849
GOTERM_BF	GO:004561	regulatic	5	1.607717	0.034843	266	63	13528	4.036281	1	0.320775	45.849
GOTERM_BF	GO:003166	cellular	5	1.607717	0.036615	266	64	13528	3.973214	1	0.331466	47.54263
GOTERM_BF	GO:00321(	positive	5	1.607717	0.036615	266	64	13528	3.973214	1	0.331466	47.54263
GOTERM_BF	GO:00068(	oxygen ar	5	1.607717	0.042236	266	67	13528	3.795309	1	0.360765	52.59236
GOTERM_BF	GO:004666	female se	5	1.607717	0.046239	266	69	13528	3.6853	1	0.377837	55.90542
GOTERM_BF	GO:004654	developme	5	1.607717	0.046239	266	69	13528	3.6853	1	0.377837	55.90542
GOTERM_BF	GO:003134	positive	5	1.607717	0.054858	266	73	13528	3.483366	1	0.414313	62.31208
GOTERM_BF	GO:000176	morphoger	5	1.607717	0.057139	266	74	13528	3.436293	1	0.421901	63.8548
GOTERM_BF	GO:00003(	response	5	1.607717	0.05947	266	75	13528	3.390476	1	0.425303	65.36991
GOTERM_BF	GO:004424	cellular	5	1.607717	0.061852	266	76	13528	3.345865	1	0.435553	66.85561
GOTERM_BF	GO:004858	developme	5	1.607717	0.091258	266	87	13528	2.922824	1	0.54194	80.89288
GOTERM_BF	GO:000193	positive	5	1.607717	0.097212	266	89	13528	2.857143	1	0.556186	82.94639
GOTERM_BF	GO:003033	positive	5	1.607717	0.097212	266	89	13528	2.857143	1	0.556186	82.94639
GOTERM_BF	GO:003134	regulatic	5	1.607717	0.097212	266	89	13528	2.857143	1	0.556186	82.94639
GOTERM_BF	GO:005191	negative	4	1.286174	1.42E-04	266	6	13528	33.90476	0.258846	0.004461	0.245822
GOTERM_BF	GO:004647	glycosphi	4	1.286174	2.45E-04	266	7	13528	29.06122	0.403477	0.007353	0.4236

GOTERM_BF	GO:001937	glycolipi	4	1.286174	2.45E-04	266	7	13528	29.06122	0.403477	0.007353	0.4236
GOTERM_BF	GO:005191	regulatic	4	1.286174	5.72E-04	266	9	13528	22.60317	0.700161	0.015324	0.984814
GOTERM_BF	GO:004668	response	4	1.286174	0.001434	266	12	13528	16.95238	0.951264	0.029471	2.451944
GOTERM_BF	GO:004273	fibrinoly	4	1.286174	0.001434	266	12	13528	16.95238	0.951264	0.029471	2.451944
GOTERM_BF	GO:000602	glycosami	4	1.286174	0.002305	266	14	13528	14.53061	0.992235	0.04172	3.913103
GOTERM_BF	GO:004369	reverse c	4	1.286174	0.003445	266	16	13528	12.71429	0.9993	0.05971	5.794611
GOTERM_BF	GO:000600	fructose	4	1.286174	0.004124	266	17	13528	11.96639	0.999833	0.068273	6.897418
GOTERM_BF	GO:001991	lipid stc	4	1.286174	0.004877	266	18	13528	11.30159	0.999966	0.077842	8.108753
GOTERM_BF	GO:000267	regulatic	4	1.286174	0.007613	266	21	13528	9.687075	1	0.113927	12.38179
GOTERM_BF	GO:000715	heterophi	4	1.286174	0.008689	266	22	13528	9.246753	1	0.12723	14.0108
GOTERM_BF	GO:004205	regulatic	4	1.286174	0.00985	266	23	13528	8.84472	1	0.139216	15.73592
GOTERM_BF	GO:001967	NAD metab	4	1.286174	0.012431	266	25	13528	8.137143	1	0.16502	19.45459
GOTERM_BF	GO:001059	regulatic	4	1.286174	0.012431	266	25	13528	8.137143	1	0.16502	19.45459
GOTERM_BF	GO:001814	peptide c	4	1.286174	0.013852	266	26	13528	7.824176	1	0.177785	21.43655
GOTERM_BF	GO:005082	protein s	4	1.286174	0.016962	266	28	13528	7.265306	1	0.204964	25.61277
GOTERM_BF	GO:000608	cellular	4	1.286174	0.016962	266	28	13528	7.265306	1	0.204964	25.61277
GOTERM_BF	GO:000931	oligosacc	4	1.286174	0.022298	266	31	13528	6.562212	1	0.246145	32.29667
GOTERM_BF	GO:000282	positive	4	1.286174	0.022298	266	31	13528	6.562212	1	0.246145	32.29667
GOTERM_BF	GO:004224	tissue re	4	1.286174	0.024257	266	32	13528	6.357143	1	0.259574	34.605
GOTERM_BF	GO:000193	regulatic	4	1.286174	0.024257	266	32	13528	6.357143	1	0.259574	34.605
GOTERM_BF	GO:000677	fat-solub	4	1.286174	0.026306	266	33	13528	6.164502	1	0.271698	36.93996
GOTERM_BF	GO:000270	positive	4	1.286174	0.028445	266	34	13528	5.983193	1	0.286429	39.29338
GOTERM_BF	GO:000270	positive	4	1.286174	0.028445	266	34	13528	5.983193	1	0.286429	39.29338
GOTERM_BF	GO:005088	neuromusc	4	1.286174	0.030674	266	35	13528	5.812245	1	0.299812	41.65717
GOTERM_BF	GO:001921	regulatic	4	1.286174	0.032991	266	36	13528	5.650794	1	0.310419	44.02337
GOTERM_BF	GO:003134	negative	4	1.286174	0.032991	266	36	13528	5.650794	1	0.310419	44.02337
GOTERM_BF	GO:000695	acute-pha	4	1.286174	0.043142	266	40	13528	5.085714	1	0.364176	53.36193
GOTERM_BF	GO:003164	regulatic	4	1.286174	0.048736	266	42	13528	4.843537	1	0.385478	57.85947
GOTERM_BF	GO:004259	response	4	1.286174	0.048736	266	42	13528	4.843537	1	0.385478	57.85947
GOTERM_BF	GO:000664	triglycer	4	1.286174	0.051659	266	43	13528	4.730897	1	0.400802	60.04416
GOTERM_BF	GO:000269	positive	4	1.286174	0.054665	266	44	13528	4.623377	1	0.414597	62.17932
GOTERM_BF	GO:000663	acylglyce	4	1.286174	0.070904	266	49	13528	4.151603	1	0.473949	71.97283
GOTERM_BF	GO:000663	neutral l	4	1.286174	0.074384	266	50	13528	4.068571	1	0.486671	73.73436
GOTERM_BF	GO:004558	regulatic	4	1.286174	0.077939	266	51	13528	3.988796	1	0.500598	75.42558
GOTERM_BF	GO:004863	regulatic	4	1.286174	0.077939	266	51	13528	3.988796	1	0.500598	75.42558
GOTERM_BF	GO:000666	glycerol	4	1.286174	0.077939	266	51	13528	3.988796	1	0.500598	75.42558

GOTERM_BF	GO:00090€	glutamine	4	1.286174	0.081567	266	52	13528	3.912088	1	0.512911	77.04546
GOTERM_BF	GO:00189(	organic €	4	1.286174	0.085267	266	53	13528	3.838275	1	0.525011	78.59342
GOTERM_BF	GO:00100(	glial cel	4	1.286174	0.085267	266	53	13528	3.838275	1	0.525011	78.59342
GOTERM_BF	GO:004247	odontoger	4	1.286174	0.089038	266	54	13528	3.767196	1	0.535499	80.06927
GOTERM_BF	GO:00515€	response	4	1.286174	0.092877	266	55	13528	3.698701	1	0.545786	81.47322
GOTERM_BF	GO:00028Ž	regulatic	4	1.286174	0.092877	266	55	13528	3.698701	1	0.545786	81.47322
GOTERM_BF	GO:00080Ƹ	cell recc	4	1.286174	0.092877	266	55	13528	3.698701	1	0.545786	81.47322
GOTERM_BF	GO:003294	positive	4	1.286174	0.096783	266	56	13528	3.632653	1	0.55724	82.8058
GOTERM_BF	GO:00706€	positive	4	1.286174	0.096783	266	56	13528	3.632653	1	0.55724	82.8058
GOTERM_BF	GO:00066€	gangliosi	3	0.96463	0.002235	266	4	13528	38.14286	0.990991	0.041178	3.795735
GOTERM_BF	GO:000931	oligosacc	3	0.96463	0.002235	266	4	13528	38.14286	0.990991	0.041178	3.795735
GOTERM_BF	GO:00109€	negative	3	0.96463	0.002235	266	4	13528	38.14286	0.990991	0.041178	3.795735
GOTERM_BF	GO:007061	regulatic	3	0.96463	0.005444	266	6	13528	25.42857	0.99999	0.084593	9.009141
GOTERM_BF	GO:00109€	regulatic	3	0.96463	0.005444	266	6	13528	25.42857	0.99999	0.084593	9.009141
GOTERM_BF	GO:000157	gangliosi	3	0.96463	0.009902	266	8	13528	19.07143	1	0.138966	15.81158
GOTERM_BF	GO:00029Ž	regulatic	3	0.96463	0.022167	266	12	13528	12.71429	1	0.246143	32.13909
GOTERM_BF	GO:00065Ž	arginine	3	0.96463	0.025863	266	13	13528	11.73626	1	0.269042	36.4417
GOTERM_BF	GO:00105€	negative	3	0.96463	0.029789	266	14	13528	10.89796	1	0.296517	40.72992
GOTERM_BF	GO:004671	muscle ma	3	0.96463	0.029789	266	14	13528	10.89796	1	0.296517	40.72992
GOTERM_BF	GO:00069€	complemer	3	0.96463	0.033935	266	15	13528	10.17143	1	0.31512	44.9614
GOTERM_BF	GO:00019€	regulatic	3	0.96463	0.038291	266	16	13528	9.535714	1	0.339715	49.09936
GOTERM_BF	GO:001004	response	3	0.96463	0.042847	266	17	13528	8.97479	1	0.363565	53.11261
GOTERM_BF	GO:00019Ƹ	negative	3	0.96463	0.047593	266	18	13528	8.47619	1	0.382395	56.97545
GOTERM_BF	GO:00343€	plasma li	3	0.96463	0.057621	266	20	13528	7.628571	1	0.42185	64.17299
GOTERM_BF	GO:00343€	macromole	3	0.96463	0.057621	266	20	13528	7.628571	1	0.42185	64.17299
GOTERM_BF	GO:00511€	cofactor	3	0.96463	0.057621	266	20	13528	7.628571	1	0.42185	64.17299
GOTERM_BF	GO:00343€	protein-l	3	0.96463	0.057621	266	20	13528	7.628571	1	0.42185	64.17299
GOTERM_BF	GO:004647	phosphati	3	0.96463	0.062885	266	21	13528	7.265306	1	0.438344	67.48115
GOTERM_BF	GO:005101	actin fil	3	0.96463	0.062885	266	21	13528	7.265306	1	0.438344	67.48115
GOTERM_BF	GO:00163Ƹ	calcium-c	3	0.96463	0.073871	266	23	13528	6.63354	1	0.485614	73.48136
GOTERM_BF	GO:00019(	cell kill	3	0.96463	0.079578	266	24	13528	6.357143	1	0.506726	76.17008
GOTERM_BF	GO:001074	regulatic	3	0.96463	0.085417	266	25	13528	6.102857	1	0.524258	78.65383
GOTERM_BF	GO:00217€	glial cel	3	0.96463	0.09138	266	26	13528	5.868132	1	0.541062	80.93752
GOTERM_BF	GO:00311(	organ reg	3	0.96463	0.09138	266	26	13528	5.868132	1	0.541062	80.93752
GOTERM_BF	GO:00019€	regulatic	3	0.96463	0.097462	266	27	13528	5.650794	1	0.555804	83.02791
GOTERM_BF	GO:00454Ž	regulatic	3	0.96463	0.097462	266	27	13528	5.650794	1	0.555804	83.02791

GOTERM_BF	GO:004248	positive	2	0.643087	0.038796	266	2	13528	50.85714	1	0.341999	49.55922
GOTERM_BF	GO:003461	cellular	2	0.643087	0.038796	266	2	13528	50.85714	1	0.341999	49.55922
GOTERM_BF	GO:000186	regulatic	2	0.643087	0.038796	266	2	13528	50.85714	1	0.341999	49.55922
GOTERM_BF	GO:004248	positive	2	0.643087	0.038796	266	2	13528	50.85714	1	0.341999	49.55922
GOTERM_BF	GO:000186	negative	2	0.643087	0.038796	266	2	13528	50.85714	1	0.341999	49.55922
GOTERM_BF	GO:000611	NADH oxic	2	0.643087	0.038796	266	2	13528	50.85714	1	0.341999	49.55922
GOTERM_BF	GO:004003	negative	2	0.643087	0.057628	266	3	13528	33.90476	1	0.420504	64.17749
GOTERM_BF	GO:004251	regulatic	2	0.643087	0.057628	266	3	13528	33.90476	1	0.420504	64.17749
GOTERM_BF	GO:004591	negative	2	0.643087	0.057628	266	3	13528	33.90476	1	0.420504	64.17749
GOTERM_BF	GO:004251	positive	2	0.643087	0.057628	266	3	13528	33.90476	1	0.420504	64.17749
GOTERM_BF	GO:003044	regulatic	2	0.643087	0.057628	266	3	13528	33.90476	1	0.420504	64.17749
GOTERM_BF	GO:001940	alditol c	2	0.643087	0.057628	266	3	13528	33.90476	1	0.420504	64.17749
GOTERM_BF	GO:004227	susceptib	2	0.643087	0.057628	266	3	13528	33.90476	1	0.420504	64.17749
GOTERM_BF	GO:004617	polyol ca	2	0.643087	0.076092	266	4	13528	25.42857	1	0.493373	74.55988
GOTERM_BF	GO:000700	outer mit	2	0.643087	0.076092	266	4	13528	25.42857	1	0.493373	74.55988
GOTERM_BF	GO:007009	mitochonc	2	0.643087	0.076092	266	4	13528	25.42857	1	0.493373	74.55988
GOTERM_BF	GO:004582	negative	2	0.643087	0.076092	266	4	13528	25.42857	1	0.493373	74.55988
GOTERM_BF	GO:001061	positive	2	0.643087	0.076092	266	4	13528	25.42857	1	0.493373	74.55988
GOTERM_BF	GO:000292	negative	2	0.643087	0.076092	266	4	13528	25.42857	1	0.493373	74.55988
GOTERM_BF	GO:001474	positive	2	0.643087	0.076092	266	4	13528	25.42857	1	0.493373	74.55988
GOTERM_BF	GO:006033	regulatic	2	0.643087	0.076092	266	4	13528	25.42857	1	0.493373	74.55988
GOTERM_BF	GO:003296	collagen	2	0.643087	0.094196	266	5	13528	20.34286	1	0.549726	81.93362
GOTERM_BF	GO:005100	negative	2	0.643087	0.094196	266	5	13528	20.34286	1	0.549726	81.93362
GOTERM_BF	GO:000688	cellular	2	0.643087	0.094196	266	5	13528	20.34286	1	0.549726	81.93362
GOTERM_BF	GO:007012	ciliary r	2	0.643087	0.094196	266	5	13528	20.34286	1	0.549726	81.93362
GOTERM_BF	GO:002261	membrane	2	0.643087	0.094196	266	5	13528	20.34286	1	0.549726	81.93362
GOTERM_BF	GO:004651	ceramide	2	0.643087	0.094196	266	5	13528	20.34286	1	0.549726	81.93362
GOTERM_BF	GO:004248	regulatic	2	0.643087	0.094196	266	5	13528	20.34286	1	0.549726	81.93362
GOTERM_BF	GO:003038	fructose	2	0.643087	0.094196	266	5	13528	20.34286	1	0.549726	81.93362

Supporting Information Tables 8

Sub-cellular distributions of 318 proteins in human urine

Category		Term	Count	%	PValue	List Tota	Pop Hits	Pop Total	Fold Enri	Bonferror	Benjamini	FDR
GOTERM_CC	GO:001602	membrane	150	48.23151	0.020138	278	7266	15294	1.135722	0.565737	0.073024	17.07386
GOTERM_CC	GO:004442	extracell	98	31.51125	2.17E-47	278	960	15294	5.616052	8.91E-46	8.91E-46	2.00E-44
GOTERM_CC	GO:000561	extracell	75	24.11576	2.54E-37	278	685	15294	6.023473	1.04E-35	5.20E-36	2.34E-34
GOTERM_CC	GO:003198	vesicle	45	14.46945	8.72E-14	278	670	15294	3.694996	3.58E-12	7.16E-13	8.03E-11
GOTERM_CC	GO:000026	cell frac	43	13.82637	2.20E-06	278	1083	15294	2.18432	9.03E-05	1.13E-05	0.002026
GOTERM_CC	GO:003101	extracell	36	11.57556	8.53E-17	278	345	15294	5.740632	4.55E-15	1.55E-15	9.99E-14
GOTERM_CC	GO:000998	cell surf	33	10.61093	3.66E-14	278	348	15294	5.216882	1.50E-12	3.75E-13	3.37E-11
GOTERM_CC	GO:004299	cell proj	26	8.360129	9.14E-04	278	697	15294	2.052187	0.036809	0.004158	0.838248
GOTERM_CC	GO:004517	apical pa	19	6.109325	4.27E-09	278	179	15294	5.839516	1.75E-07	2.50E-08	3.93E-06
GOTERM_CC	GO:004442	extracell	17	5.466238	3.40E-10	278	117	15294	7.993544	1.40E-08	2.33E-09	3.13E-07
GOTERM_CC	GO:003435	plasma li	5	1.607717	0.003544	278	35	15294	7.859198	0.135475	0.014452	3.214707
GOTERM_CC	GO:003299	protein-l	5	1.607717	0.003544	278	35	15294	7.859198	0.135475	0.014452	3.214707

Supporting Information Tables 9. Information of patients and controls involved in urine CP analysis.

	Cancer（n=60）		Control(n=60)	
**Characteristic**	NO.	%	NO.	%
**Age Year**				
Median	54.2		45.1	
Range	46-73		25-78	
**Sex**				
Female	22	36.6%	25	41.6%
Male	38	63.4%	35	58.4%
**Smoking**	26	43.3%	28	46.7%
**TNM stage**				
T1N0M0	15	25.0%	--	--
T2N0M0	21	35.0%	--	--
T2N0M1	1	1.6%	--	--
T2N1M0	11	18.3%	--	--
T2N2M0	8	13.3%	--	--
T3N0M0	1	1.6%	--	--
T3N1M0	1	1.6%	--	--
T4N0M0	1	1.6%	--	--
T4N2M0	1	1.6%	--	--
**Histology**			--	--
Squamous cell Carcinoma	25	41.6%	--	--
Adneocarcinoma	29	48.3%	--	--
Adneosquamous carcinoma	2	3.3%	--	--
Large cell carcinoma	1	1.6%	--	--
Small cell lung cancer	2	3.3%	--	--
Bronchioloalveolar carcinoma	1	1.6%	--	--

Supporting Information Tables 10. Information of patients and controls involved in urine Orm1 analysis.

	Cancer（n=16）		Control(n=8)	
**Characteristic**	NO.	%	NO.	%
**Age Year**				
Median	48.9		56.6	
Range	22-64		44-75	
**Sex**				
Female	4	25.0%	3	37.5%
Male	12	75.0%	5	62.5%
**Smoking**	7	43.7%	4	50%
**Histology**				
Squamous cell Carcinoma	2	12.5%	--	--
Adenocarcinoma	13	81.3%	--	--
Small cell lung cancer	1	6.3%	--	--
